# PIC-SURE: an open-source platform for integrating clinical and genomic data

**DOI:** 10.1038/s41746-025-02284-9

**Published:** 2025-12-30

**Authors:** Alba Gutiérrez-Sacristán, Emily E. Hughes, Ryan Amari, George Colon, Thomas N. DeSain, Sharra Neely, James T. Peck, Samantha Piatt, Lucas Sikina, Jessica Lyons, Danielle Pillion, Paul Avillach

**Affiliations:** 1 Department of Biomedical Informatics, Harvard Medical School, 10 Shattuck Street, Boston, MA USA; 2 Computational Health Informatics Program, Boston Children’s Hospital, 300 Longwood Avenue, Boston, MA USA

**Keywords:** Computational biology and bioinformatics, Genetics

## Abstract

PIC-SURE is an open-source platform for integrating and analyzing large-scale clinical and genomic data. Part of the NIH NHLBI BioData Catalyst® ecosystem, PIC-SURE enables real-time cohort building and analysis across 1.4 M participants within 273 studies (May 2025). Through a graphical interface and API, researchers can explore and analyze complex datasets in real time. This flexibility supports scalable and reproducible research, lowering the barrier to integrated clinical and genomic data analysis.

## Background and rationale

As biomedical datasets grow in volume and diversity, so do the opportunities for advancing precision medicine by effectively leveraging clinical and genomic data^1,2^. However, as the datasets grow, so do the challenges around scaling data access and integration, managing heterogeneity, and ensuring reproducibility and efficient analysis.

Many platforms^3,4^ support the clinical data extraction and multimodal data analysis. But most existing platforms have limited capacity for large-scale genomic integration and are often limited to single-nucleotide polymorphism (SNP) level without support for whole-genome sequencing (WGS)^5^. These limitations are further compounded by the need to manage terabyte-scale datasets, enforce user- and consent-specific access, and maintain data privacy and security^6^.

### PIC-SURE: architecture and capabilities

**Fig. 1:**
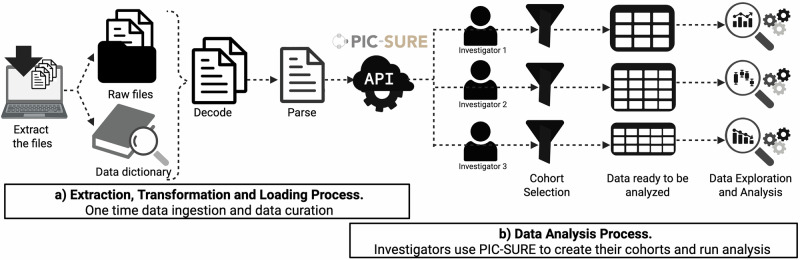
PIC-SURE data analysis workflow. **a** ETL process: data are extracted, transformed, and loaded once, enabling access via the PIC-SURE interface and application programming interface (API). **b** Data analysis process: Researchers identify cohorts based on research questions through the PIC-SURE User Interface (UI)/ Application Programming Interface (API). Results are returned as an analysis-ready table. Figure created with BioRender.com.

To address these challenges, we developed PIC-SURE (Patient-centered Information Commons: Standard Unification of Research Elements), a streamlined, user-friendly platform that enables researchers to access and analyze integrated clinical and genomic data for cohort identification, hypothesis testing, and exploratory analysis. As part of the NIH National Heart, Lung, and Blood Institute’s (NHLBI) BioData Catalyst® (BDC) ecosystem^7,8^, PIC-SURE simplifies access to large, heterogeneous datasets, empowering researchers to explore data efficiently and conduct scalable, reproducible analyses. PIC-SURE enables users to build cohorts and retrieve participant-level tables, which are made ready for analysis by integrating diverse biomedical data and exposing variable-level information. The platform provides a graphical user interface (GUI) and an application programming interface (API), supporting multi-parameter queries across clinical and genomic variables. PIC-SURE operates within a secure, cloud-based environment, adheres to the NIST 800-53 v4 framework, and holds FISMA Moderate Authority to Operate (ATO), ensuring compliance with data privacy and security standards (*Methods*). By managing the underlying data complexity—including consent group authorization, query optimization, and variable-level access—PIC-SURE lowers the technical barrier for investigators and enables rapid, reproducible research (Fig. 1). PIC-SURE preserves the original structure and metadata of each contributing study, exposing variables exactly as they were collected, rather than harmonizing them across datasets. This approach maintains full provenance and transparency, enabling researchers to inspect study-specific definitions, coding systems, and metadata through the API while still benefiting from a unified access layer that supports FAIR-compliant data discovery and retrieval. When harmonized phenotypes are available from upstream initiatives, such as the TOPMed Data Coordinating Center (DCC) cross-study harmonization project^9,10^, PIC-SURE ingests and exposes those harmonized variables alongside study-native data, enabling cross-study analyses using standardized phenotypes.

### Available data and access tiers

**Fig. 2:**
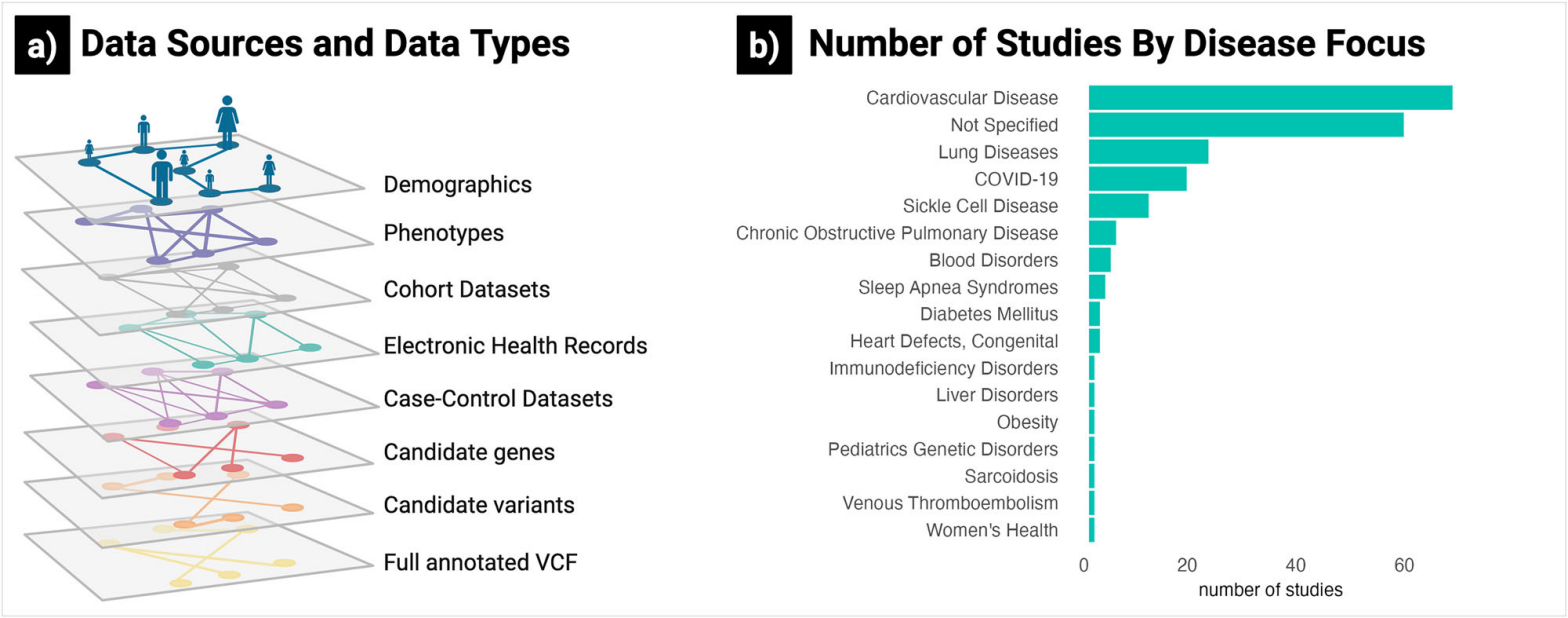
Overview of data in BioData Catalyst (BDC) Powered by PIC-SURE as of May 2025. **a** Data sources and data types: BDC PIC-SURE integrates various sources and types at the participant level. **b** Number of studies by disease focus: Disease focus of data available in BDC PIC-SURE was extracted from dbGaP and reclassified. “Not Specified” was assigned when no focus was provided. Figure created with BioRender.com.

As of May 2025, PIC-SURE provides access to participant-level data from 1,422,965 participants across 273 studies (Supplementary Table 1). These counts reflect the full BDC PIC-SURE resource and are discoverable at the aggregate level through Open PIC-SURE, while participant-level access is governed by dataset-specific permissions in the Authorized PIC-SURE tier. These include studies from the Trans-Omics for Precision Medicine (TOPMed) program^11,12^, which generates whole-genome sequencing (WGS) data linked to rich clinical phenotypes. The studies also cover a broad range of clinical areas, including cardiovascular and lung diseases (Fig. 2b), and encompass various designs, from prospective cohorts to interventional trials. PIC-SURE operates in two tiers. Open PIC-SURE enables any user—no login or authorization required—to search for clinical variables and retrieve aggregate counts for feasibility analyses. Authorized PIC-SURE allows approved users to securely query and prepare participant-level data for analysis, including genomic filtering (Supplementary Table 2). Both tiers enable rapid, privacy-preserving exploration and filtering, allowing researchers to generate analysis-ready cohorts without data downloading.

PIC-SURE’s flexible query system allows users to apply multiple filters across clinical and genomic variables, integrating data from diverse sources while preserving privacy. Researchers can explore datasets through a GUI or API, making the data accessible to users with different technical backgrounds. To support reproducibility and usability, explanatory R and Python Jupyter Notebooks are publicly available on GitHub^13^. These notebooks provide examples of real-world cohort creation, facilitating reuse and adaptation for similar research questions.

### Demonstrations of use and reproducibility

To illustrate the functionality of PIC-SURE, we utilized Open PIC-SURE to examine the co-occurrence of obesity and asthma in a patient cohort. A keyword search for “obesity” returned results from 15 studies. Specifically, the Women’s Health Initiative (WHI)^14^ yielded more than 50,000 individuals after filtering for participants with a body mass index (BMI) greater than 30. Adding a second filter for asthma refined the cohort to 5,114 participants with both conditions. This initial query can be performed in both the open and the authorized versions of PIC-SURE. When working in the authorized tier with access to a specific dataset such as WHI, the cohort can be further refined by adding genomic criteria. For example, we identified the number of individuals with asthma and obesity who carry a variant in the *FTO* gene, which is known to be associated with obesity^15^. This additional genomic filter resulted in a cohort of 370 participants. PIC-SURE enables the user to filter not only by gene, but also by variant severity, consequence, and frequency. The genomic filter can be adjusted to identify participants with obesity, asthma, and high-severity variants on the *FTO* gene, resulting in a cohort of 50 participants. This query process—completed in seconds—demonstrates PIC-SURE’s ability to generate analysis-ready cohorts and rapidly explore comorbidities.

PIC-SURE also aligns with FAIR (Findable, Accessible, Interoperable, and Reusable) principles to promote reproducibility in biomedical research by enabling transparent, well-documented access to integrated datasets. For example, researchers successfully replicated the results from the ORCHID clinical trial focused on COVID-19 by extracting and analyzing the ORCHID data using the PIC-SURE API. The full code for reproducing the analysis completed within the Serret-Larmande et al. ORCHID publication is publicly available in the PIC-SURE API GitHub repository^16^. The ability to export data in formats compatible with standard statistical tools further supports reproducible and extensible downstream analyses.

In addition to the cohort creation example using the PIC-SURE UI, the PIC-SURE API supports fully reproducible end-to-end analytical workflows. A collection of public R and Python notebooks demonstrates complete pipelines, from constructing queries and exporting data to performing downstream analyses, using real datasets available through BDC PIC-SURE. For example, an end-to-end phenotype-phenotype association analysis notebook^17,18^ illustrates cohort definition, data extraction, and statistical analysis using the PIC-SURE API, while a harmonization notebook^19,20^ demonstrates how study-specific variables can be programmatically retrieved, transformed, and analyzed. These and other workflows are openly available in the PIC-SURE API repository, providing transparent examples of how the platform enables reproducible research. Coding examples are executable for any researcher with the applicable authorization to access participant-level data.

## Usage and future directions

PIC-SURE’s architecture is built to handle datasets of varying sizes and complexity, from small feasibility studies to large-scale genomic datasets. Between January 2024 and May 2025, the platform has been utilized by over 2,000 researchers across 35 countries, resulting in more than 37,000 authorized and 33,500 open search events. Future enhancements are planned to include more advanced search capabilities and filtering options, supporting a broader range of research needs. Overall, PIC-SURE combines large-scale, heterogeneous data with real-time querying to support reproducible research across a wide range of precision medicine studies.

## Methods

### PIC-SURE high-performance data store (HPDS)

PIC-SURE-HPDS is an open-source Java database, operating under the Apache 2.0 license. The database runs in a containerized Spring application and is accessed by PIC-SURE via a REST API. PIC-SURE-HPDS is designed to support use cases in biomedical informatics without requiring massive clustering as the datasets increase in scale. PIC-SURE aims to support performant cohort construction through sub-second COUNT queries, while also allowing resource-light data export.

HPDS PIC-SURE is deployed on a cloud-native architecture using AWS EC2, with compute and storage resources scaled as datasets are added or expanded. The instance class, CPU, and RAM are adjusted to match the ingestion volume and query demand. As of May 2025, the BDC PIC-SURE instance was running on an m5.12xlarge (48 vCPUs, 192 GiB RAM) with a gp3 EBS volume provisioned at 1000 GiB, 3000 IOPS, and 125 MB/s throughput. This configuration is updated as new studies are onboarded.

**Fig. 3:**
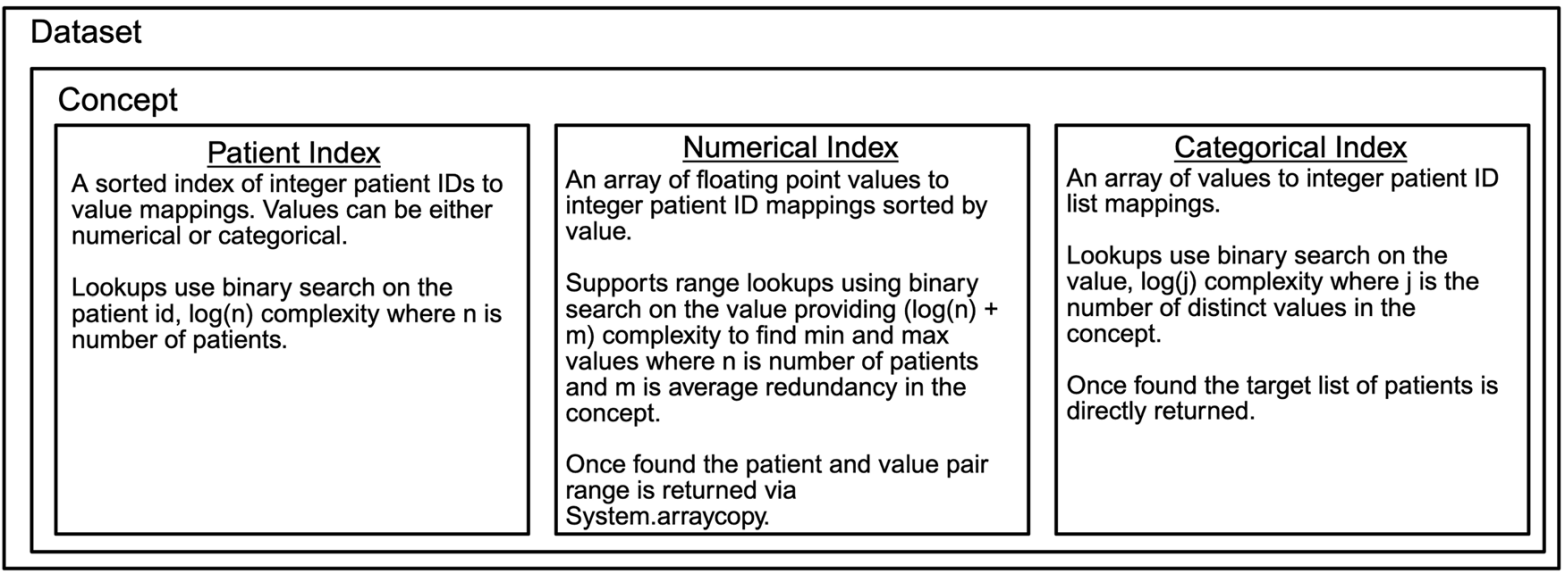
Concepts included in the data file in PIC-SURE High-Performance Data Store: patient, numerical, and categorical indices. Figure created with BioRender.com.

Clinical data datasets are stored as two files: metadata and data. The metadata file contains the internal data dictionary, high-level dataset-specific information, and file offsets for each variable’s data within the data file. The data file includes data for each of the three concepts in the layout below (Fig. 3).

When filtering patient cohorts by variable values, the Numerical Index or Categorical Index is used to obtain the list of patients who meet the filter criteria expressed in the query. The Patient Indices are optimized only when generating these filtered lists of patient identifiers with each query.

When exporting data, a list of patient identifiers is created for the filtered cohort. Then, the Patient Index is used to search for the values for each selected concept in the data export. The Numerical and Categorical indices are queried using that collection of identifiers to generate the exported data. This generation process utilizes temporary files to minimize memory overhead during export. This process is slower than a more traditional approach due to the limitation of disk IO. Still, it is also far less memory-intensive, as the query response is only stored on the disk.

While disk space is among the more affordable resources available in contemporary computation environments, there is still value in finding mechanisms to reduce the at-rest database size. When using PIC-SURE, the storage of each variable on disk is compressed using the JVM-provided GZIP implementation and then encrypted using the JVM-provided AES/GCM/NoPadding cipher instance. GZIP compression presents a powerful mechanism for space savings in HPDS. Even in PIC-SURE environments with millions of patients and thousands of genomic samples, database sizes remain well under 200 gigabytes. Keeping at-rest data sizes small allows PIC-SURE to be more cost-effective and portable. Because of these efforts, PIC-SURE instances can be cloned and modified with lower operations overhead.

Similar to a variant call format (VCF), patient genomic data is represented as the differences between the patient and the reference genome. A variant that is not represented means that all patients contain only the reference allele. Instead of storing zygosity as strings (i.e., “0/0”, “0/1”, “1/1”), the variable is stored as a bitmask value (using Java BigInteger) where each bit represents a patient and their zygosity. For example, 100100000100 represents patients 0, 3, and 9 having a zygosity. Only two to four states of zygosity are stored: heterozygous, homozygous, and optionally heterozygous no-call and homozygous no-call. This process results in a worst-case storage requirement of (*n* x 4) bits for each variant, where *n* is the number of samples in the dataset. Storage is further optimized for sparsely populated variants (i.e., variants present in only a few patients) by storing them as a set of matching patient identifiers. Because the variant information is stored as integers in the sparse scenario, the storage for these variants is (*n* x 4) bytes, where *n* is the number of patients with a variant.

Additionally, this bitmask-based variant storage is optimized to filter patients with minimal additional processing or translation. To filter a list of variants, a single bitwise OR operation is required for each variant involved in the query. The offset of 1 value in the resulting bitmask corresponds to the offset in an int[] containing the patient identifiers, with at least one variant in the list of filtered variants. The BigInteger class internally stores these bitmasks as an int[] and orchestrates the bitwise ORs across the values in this int[].

Variant annotations are stored using the same Numerical Index and Categorical Index described above, indexing variant IDs instead of patient identifiers. Each record in the storage file is a list of integers referencing the specification of each variant in the system. The variant specification is of the form <chrom > ,<offset > ,<ref > ,<alt > ,<gene > ,<consequence > . The variant identifier value corresponds to the offset in this array of the String representation of the variant. These representations are translated into BigInteger bitmasks in-memory, and bitwise AND/OR operations are used to calculate variants specified by a query.

To enable parallelization of computation, the genomic data can be partitioned by both chromosomes and patient sets. This partitioning is slightly less efficient in terms of storage but allows calculations to be done in parallel on smaller chunks of data. This partitioning is possible on a single filesystem or distributed across multiple servers.

### PIC-SURE API

The PIC-SURE API is an Apache 2.0 open-source software that fosters the incorporation of multiple heterogeneous patient-level clinical, omics, and environmental datasets. The software embraces the concept of decentralized datasets of varying types while maintaining a simple communication layer that facilitates querying, joining, and computation.

The first step in the data flow process is organizing the input data into Variant Call Format (VCF) files for genomic information and Comma-Separated Value (CSV) files for non-genomic data. (For more information about the genomic annotation process, refer to the Genomic Reference Build and Annotation Pipeline section below.) The second step is an ETL (Extract, Transform, Load) process, which enables the synthesis and accommodation of diverse data types before they are exposed through PIC-SURE. Next, the data is ready to be queried using the user interface (UI)and the API. Users can access the data from the search portal UI and the R or Python adapters of the API. These PIC-SURE tools bypass the need to download the entire dataset or process the VCF files. Through queries, investigators can verify multiple hypotheses and narrow the original cohort to a subset of interest.

PIC-SURE enables investigators to transition seamlessly from one layer of information to another. The search portal functions as a search engine, and investigators can enter any term of interest (e.g., asthma, sex)and select the variable’s specific value (e.g., positive, Female). Additional filters can be applied to further refine the query.

Researchers can also combine phenotypic and genomic filtering in a single query by adding a genomic filter. This function enables investigators to create a comprehensive genomic filter using the gene with variant, variant frequency, and variant consequence grouped by severity. For example, investigators could refine their query to include participants with a high-severity variant in the *CHD8* gene. Investigators could also filter by family history, restricting their cohort to those with affected relatives.

Once all the filters have been applied, any additional variables of interest can be added to the export without needing to apply any further filters. Clicking “Prepare Data for Analysis” provides a tabular summary of the query. From here, the investigator can review their query, export the data to an analysis workspace, and save the Dataset ID associated with the query to reproduce the query at any time and for further analysis using the PIC-SURE API. Results will always include a participant identifier and the consent information, independent of the variables filtered and selected.

### Genomic reference build and annotation pipeline

Genomic data ingested into the PIC-SURE High-Performance Data Store (HPDS) are aligned and harmonized to the GRCh38 reference genome. Variant annotation is performed using the Ensembl Variant Effect Predictor (VEP)^21^ following the HPDS annotation workflow available at: https://github.com/bch-gnome/hpds_annotation. The pipeline supports single-nucleotide variants (SNVs), short insertions and deletions (indels), and multi-allelic variants, which are decomposed and normalized prior to annotation. VEP generates functional consequence annotations for all alleles, including both coding and non-coding effects (e.g., intronic, UTR, and regulatory region annotations). Applicable VEP outputs described in the annotation pipeline are stored in HPDS and accessible through both the PIC-SURE API and graphical user interface for authorized users.

### Consequence severity groupings

Variant consequences are grouped using Ensembl’s standard impact categories: high impact, moderate impact, low impact, and modifier^22^. These severity groupings support genomic filtering within PIC-SURE, enabling users to refine cohorts based on variant consequences aggregated by impact category.

### PIC-SURE security design

To sustain projects across diverse resource types, PIC-SURE API focuses on flexibility. The PIC-SURE API Resource Interfaces accept queries in the native representation of each backing resource, wrapped in a thin layer of routing. Results are passed through unmodified from the resource to the user without intermediate translations or storage. The security model does not assume how a resource stores its data. Instead of determining if a user may interact with a specific piece of data, the system asserts how the query is structured and what it can or must contain based on Access Rules.

PIC-SURE Auth Micro-App (PSAMA) determines whether a user can query data, as indicated by the Access Rules assigned to that user. This Access Rule abstraction can also be configured to enable electronic health record (EHR) use cases. For example, a user may retrieve aggregate data for any query and receive more explicit authorization for patient-level data and multi-study environments. There, specific studies, variables, or participants are authorized for each user. The PIC-SURE platform adheres to the NIST 800-53 v4 framework and has a FISMA Moderate ATO.

Additional information about PIC-SURE and how to deploy it can be found on GitHub: https://github.com/hms-dbmi/pic-sure-all-in-one under Apache License 2.0. The repository for the Python adapter of the PIC-SURE API is located at https://github.com/hms-dbmi/pic-sure-python-client, and the R adapter is located at https://github.com/hms-dbmi/pic-sure-r-client. The PIC-SURE User Guide can be found at https://pic-sure.gitbook.io/nhlbi-biodata-catalyst-powered-by-pic-sure.

### Ethics statement

This study used only existing, de-identified data within the NIH NHLBI BioData Catalyst® ecosystem and did not involve direct experimentation on human participants or animals. All data were used in accordance with NIH data-use and privacy policies. The research protocol was reviewed and approved by the Harvard Medical School Institutional Review Board (HMS IRB) under IRB Protocol number IRB18-0039.

### Consent to participate

No new human participants were recruited for this study. The analyses were conducted using existing, de-identified data from studies available through the NIH NHLBI BioData Catalyst® ecosystem. All participants provided informed consent as part of the original studies from which the data were derived. The dbGaP data are organized into consent groups that define the permitted uses of each dataset, and this study adhered strictly to those data-use limitations and institutional review requirements. The approval in order to obtain access to data via dbGaP was reviewed and approved by the Harvard Medical School Institutional Review Board (HMS IRB). As this work involved only infrastructure development using de-identified data, no additional consent was required.

## Supplementary information

SupplementaryMaterial.

## Data Availability

Data availability The data is available through NHLBI BioData Catalyst® Powered by PIC-SURE and dbGaP. Aggregated summary statistics can be accessed directly through the BDC-PIC-SURE interface (https://picsure.biodatacatalyst.nhlbi.nih.gov/). To access participant-level data, a Data Access Request must be submitted for each study through dbGaP (https://dbgap.ncbi.nlm.nih.gov/aa).

## Code availability

Instructions and code to deploy PIC-SURE are publicly available on GitHub under an Apache License 2.0: https://github.com/hms-dbmi/pic-sure-all-in-one.
