# Supplementary material for: PIC-SURE: an open-source platform for integrating clinical and genomic data
Source: NPJ Digit Med. 2025 Dec 30;9:96. doi: 10.1038/s41746-025-02284-9 (PMC12855934; doi:10.1038/s41746-025-02284-9)
Supplement: Supplementary file 1 — SupplementaryMaterial. [file 41746_2025_2284_MOESM1_ESM.docx]

**Supplementary Material**

**Supplementary Table 1. Studies included in BDC PIC-SURE**

| **Abbreviation** | **Name** | **Study Focus** | **Study Type** | **Participants** | **Clinical Variables** | **Study Link** |
| --- | --- | --- | --- | --- | --- | --- |
| 1000Genomes | IGSR: The International Genome Sample Resource | N/A | Population-based Cohort | 4978 | 26 | [open_access-1000Genomes](https://www.internationalgenome.org/) |
| AACAC | NHLBI TOPMed: Diabetes Heart Study (DHS) African American Coronary Artery Calcification (AA CAC) | Cardiovascular Diseases | Cross-Sectional | 3 | 54 | [phs001412.v3.p1.c2](https://www.ncbi.nlm.nih.gov/projects/gap/cgi-bin/study.cgi?study_id=phs001412.v3.p1) |
| AACAC | NHLBI TOPMed: Diabetes Heart Study (DHS) African American Coronary Artery Calcification (AA CAC) | Cardiovascular Diseases | Cross-Sectional | 402 | 54 | [phs001412.v3.p1.c1](https://www.ncbi.nlm.nih.gov/projects/gap/cgi-bin/study.cgi?study_id=phs001412.v3.p1) |
| ACTIV-4_Host_Tissue | CONNECTS Master Protocol for Clinical Trials targeting Macro- and Micro-Immuno-Thrombosis, Vascular Hyperinflammation, and Hypercoagulability and Renin-Angiotensin-Aldosterone System (RAAS) in Hospitalized Patients with COVID-19 (ACTIV-4 Host Tissue) | COVID-19 Drug Treatment | Clinical Trial | 899 | 682 | [phs003708.v2.p2.c1](https://www.ncbi.nlm.nih.gov/projects/gap/cgi-bin/study.cgi?study_id=phs003708.v2.p2) |
| ACTIV4a | COVID-19 ACTIV-4 ACUTE: A Multicenter, Adaptive, Randomized Controlled Platform Trial of the Safety and Efficacy of Antithrombotic Strategies in Hospitalized Adults with COVID-19 (ACTIV4A) | COVID-19 | Interventional | 3425 | 333 | [phs002694.v4.p1.c1](https://www.ncbi.nlm.nih.gov/projects/gap/cgi-bin/study.cgi?study_id=phs002694.v4.p1) |
| ACTIV4b | COVID-19 Positive Outpatient Thrombosis Prevention in Adults Aged 40-80 | COVID-19 | Interventional | 657 | 311 | [phs002710.v1.p1.c1](https://www.ncbi.nlm.nih.gov/projects/gap/cgi-bin/study.cgi?study_id=phs002710.v1.p1) |
| ACTIV4c | COVID-19: Post-Hospital Thromboprophylaxis A Multicenter, Adaptive, Prospective, Randomized Trial Evaluating the Efficacy and Safety of Antithrombotic Strategies in Patients with COVID-19 Following Hospital Discharge (ACTIV-4C) | COVID-19 | Interventional | 1217 | 594 | [phs003063.v1.p1.c1](https://www.ncbi.nlm.nih.gov/projects/gap/cgi-bin/study.cgi?study_id=phs003063.v1.p1) |
| AFLMU | NHLBI TOPMed: NHGRI CCDG: AF Biobank LMU in the context of the MED Biobank LMU | Atrial Fibrillation | Case Set | 350 | 18 | [phs001543.v3.p1.c1](https://www.ncbi.nlm.nih.gov/projects/gap/cgi-bin/study.cgi?study_id=phs001543.v3.p1) |
| AMISH | NHLBI TOPMed: Genetics of Cardiometabolic Health in the Amish | Cardiovascular Disease | Family/Twin/Trios | 1123 | 74 | [phs000956.v5.p1.c2](https://www.ncbi.nlm.nih.gov/projects/gap/cgi-bin/study.cgi?study_id=phs000956.v5.p1) |
| ARIC | Atherosclerosis Risk in Communities (ARIC) - The Collaborative Cohort of Cohorts for COVID-19 Research (C4R) | COVID-19 | Prospective Longitudinal Cohort (clinical data) and Case-Control (genomic data) | 361 | 265 | [phs002988.v1.p1.c2](https://www.ncbi.nlm.nih.gov/projects/gap/cgi-bin/study.cgi?study_id=phs002988.v1.p1) |
| ARIC | Atherosclerosis Risk in Communities (ARIC) - The Collaborative Cohort of Cohorts for COVID-19 Research (C4R) | COVID-19 | Prospective Longitudinal Cohort (clinical data) and Case-Control (genomic data) | 6162 | 265 | [phs002988.v1.p1.c1](https://www.ncbi.nlm.nih.gov/projects/gap/cgi-bin/study.cgi?study_id=phs002988.v1.p1) |
| ARIC | NHLBI TOPMed - NHGRI CCDG: Atherosclerosis Risk in Communities (ARIC) | Cardiovascular Disease | Prospective Longitudinal Cohort (clinical data) and Case-Control (genomic data) | 89 | 32 | [phs001211.v5.p4.c2](https://www.ncbi.nlm.nih.gov/projects/gap/cgi-bin/study.cgi?study_id=phs001211.v5.p4) |
| ARIC | NHLBI TOPMed - NHGRI CCDG: Atherosclerosis Risk in Communities (ARIC) | Cardiovascular Disease | Prospective Longitudinal Cohort (clinical data) and Case-Control (genomic data) | 11496 | 32 | [phs001211.v5.p4.c1](https://www.ncbi.nlm.nih.gov/projects/gap/cgi-bin/study.cgi?study_id=phs001211.v5.p4) |
| AustralianFamilialAF | NHLBI TOPMed: Australian Familial Atrial Fibrillation Study | Atrial Fibrillation | Case Set | 120 | 31 | [phs001435.v2.p1.c1](https://www.ncbi.nlm.nih.gov/projects/gap/cgi-bin/study.cgi?study_id=phs001435.v2.p1) |
| BABYHUG | Hydroxyurea to Prevent Organ Damage in Children with Sickle Cell Anemia (BABY HUG) Phase III Clinical Trial and Follow-Up Observational Studies I and II | Anemia, Sickle Cell | Clinical Trial | 219 | 2637 | [phs002415.v1.p1.c1](https://www.ncbi.nlm.nih.gov/projects/gap/cgi-bin/study.cgi?study_id=phs002415.v1.p1) |
| BAGS | NHLBI TOPMed: The Genetics and Epidemiology of Asthma in Barbados | Asthma | Family/Twin/Trios | 1527 | 54 | [phs001143.v4.p1.c1](https://www.ncbi.nlm.nih.gov/projects/gap/cgi-bin/study.cgi?study_id=phs001143.v4.p1) |
| BioLINCC_ARDSNet-ALTA | Acute Respiratory Distress Network (ARDSNet) Studies 06 and 08 Prospective, Randomized, Multicenter Trial of Aerosolized Albuterol Versus Placebo for the Treatment of Acute Lung Injury (ALTA) (ARDSNet-ALTA-BioLINCC) |  | Clinical Trial | 282 | 781 | [phs003743.v1.p1.c1](https://www.ncbi.nlm.nih.gov/projects/gap/cgi-bin/study.cgi?study_id=phs003743.v1.p1) |
| BioLINCC_ARDSNet-ALVEOLI | Acute Respiratory Distress Network (ARDSNet) Study 04 Assessment of Low Tidal Volume and Elevated End-Expiratory Volume to Obviate Lung Injury (ALVEOLI-BioLINCC) |  | Clinical Trial | 550 | 223 | [phs003714.v1.p1.c1](https://www.ncbi.nlm.nih.gov/projects/gap/cgi-bin/study.cgi?study_id=phs003714.v1.p1) |
| BioLINCC_ARDSNet-LASRS | Acute Respiratory Distress Network (ARDSNet) Study 02 Late Steroid Rescue Study (LaSRS) |  | Clinical Trial | 180 | 154 | [phs003769.v1.p1.c1](https://www.ncbi.nlm.nih.gov/projects/gap/cgi-bin/study.cgi?study_id=phs003769.v1.p1) |
| BioLINCC_ARDSNet-Omega | Acute Respiratory Distress Network (ARDSNet) Studies 07 and 08 Prospective, Randomized, Blinded, Placebo-controlled, Multi-center Trial of Omega-3 Fatty Acid, Gamma-Linolenic Acid, and Anti-Oxidant Supplementation in the Management of Acute Lung Injury |  | Clinical Trial | 272 | 829 | [phs003744.v1.p1.c1](https://www.ncbi.nlm.nih.gov/projects/gap/cgi-bin/study.cgi?study_id=phs003744.v1.p1) |
| BioLINCC_ARDSNet-SAILS | Acute Respiratory Distress Network (ARDSNet) Studies 10 and 12 Statins for Acutely Injured Lungs from Sepsis (SAILS) (ARDSNet-SAILS-BioLINCC) |  | Clinical Trial | 745 | 1075 | [phs003736.v1.p1.c1](http://www.ncbi.nlm.nih.gov/projects/gap/cgi-bin/study.cgi?study_id=phs003736.v1.p1) |
| BioLINCC_ARMA-KARMA-LARMA | Acute Respiratory Distress Network (ARDSNet) Studies 01 and 03 Lower Versus Higher Tidal Volume, Ketoconazole Treatment and Lisofylline Treatment (ARMA/KARMA/LARMA) (ARDSNet-ARMA/KARMA/LARMA-BioLINCC) |  | Clinical Trial | 902 | 18 | [phs003734.v1.p1.c1](http://www.ncbi.nlm.nih.gov/projects/gap/cgi-bin/study.cgi?study_id=phs003734.v1.p1) |
| BioLINCC_BEST | Beta-Blocker Evaluation in Survival Trial (BEST-BioLINCC) |  | Clinical Trial | 2707 | 416 | [phs003730.v1.p1.c1](https://www.ncbi.nlm.nih.gov/projects/gap/cgi-bin/study.cgi?study_id=phs003730.v1.p1) |
| BioLINCC_BEST_CLI | Best Endovascular vs. Best Surgical Therapy in Patients With Critical Limb Ischemia |  | Clinical Trial | 1830 | 256 | [phs003844.v1.p1.c1](https://www.ncbi.nlm.nih.gov/projects/gap/cgi-bin/study.cgi?study_id=phs003844.v1.p1) |
| biolincc_camp | Childhood Asthma Management Program (CAMP): Dataset for Teaching Purposes | Asthma | Clinical Trial - Training Dataset | 695 | 28 | [tutorial-biolincc_camp](https://biolincc.nhlbi.nih.gov/teaching/) |
| BioLINCC_CARDIA | Coronary Artery Risk Development in Young Adults (CARDIA) BioLINCC |  | Prospective Longitudinal Cohort | 4411 | 13422 | [phs003739.v1.p1.c1](https://www.ncbi.nlm.nih.gov/projects/gap/cgi-bin/study.cgi?study_id=phs003739.v1.p1) |
| BioLINCC_CARDIA | Coronary Artery Risk Development in Young Adults (CARDIA) BioLINCC |  | Prospective Longitudinal Cohort | 133 | 13422 | [phs003739.v1.p1.c2](https://www.ncbi.nlm.nih.gov/projects/gap/cgi-bin/study.cgi?study_id=phs003739.v1.p1) |
| BioLINCC_DIG | Digitalis Investigation Group (DIG) |  | Clinical Trial | 7788 | 49 | [phs003872.v1.p1.c1](https://www.ncbi.nlm.nih.gov/projects/gap/cgi-bin/study.cgi?study_id=phs003872.v1.p1) |
| biolincc_digitalis | Digitalis Investigation Group (DIG) Trial : Dataset for Teaching Purposes |  | Clinical Trial - Training Dataset | 6800 | 72 | [tutorial-biolincc_digitalis](https://biolincc.nhlbi.nih.gov/teaching/) |
| BioLINCC_ESCAPE | Evaluation Study of Congestive Heart Failure and Pulmonary Artery Catheterization Effectiveness (ESCAPE) |  | Clinical Trial | 872 | 221 | [phs003782.v1.p1.c1](https://www.ncbi.nlm.nih.gov/projects/gap/cgi-bin/study.cgi?study_id=phs003782.v1.p1) |
| biolincc_framingham | Framingham Heart Study : Dataset for Teaching Purposes | Cardiovascular Disease | Prospective Longitudinal Cohort - Training Dataset | 4434 | 38 | [tutorial-biolincc_framingham](https://biolincc.nhlbi.nih.gov/teaching/) |
| BioLINCC_GUIDE-IT | Guiding Evidence Based Therapy Using Biomarker Intensified Treatment in Heart Failure (GUIDE-IT) | Heart Failure | Clinical Trial | 894 | 997 | [phs003621.v1.p1.c1](https://www.ncbi.nlm.nih.gov/projects/gap/cgi-bin/study.cgi?study_id=phs003621.v1.p1) |
| BioLINCC_HF-ACTION | Heart Failure: A Controlled Trial Investigating Outcomes of Exercise Training (HF-ACTION) | Cardiovascular Diseases | Clinical Trial | 377 | 278 | [phs003599.v1.p1.c2](https://www.ncbi.nlm.nih.gov/projects/gap/cgi-bin/study.cgi?study_id=phs003599.v1.p1) |
| BioLINCC_HF-ACTION | Heart Failure: A Controlled Trial Investigating Outcomes of Exercise Training (HF-ACTION) | Cardiovascular Diseases | Clinical Trial | 1753 | 278 | [phs003599.v1.p1.c1](https://www.ncbi.nlm.nih.gov/projects/gap/cgi-bin/study.cgi?study_id=phs003599.v1.p1) |
| BioLINCC_HFN-ATHENA | Heart Failure Network Aldosterone Targeted Neurohormonal Combined with Natriuresis Therapy - (HFN ATHENA-BioLINCC) | Heart Failure | Clinical Trial | 360 | 243 | [phs003506.v1.p1.c1](https://www.ncbi.nlm.nih.gov/projects/gap/cgi-bin/study.cgi?study_id=phs003506.v1.p1) |
| BioLINCC_HFN-CARRESS | Heart Failure Network - Effectiveness of Ultrafiltration in Treating People with Acute Decompensated Heart Failure and Cardiorenal Syndrome (HFN CARRESS - BioLINCC) |  | Clinical Trial | 188 | 279 | [phs003510.v1.p1.c1](https://www.ncbi.nlm.nih.gov/projects/gap/cgi-bin/study.cgi?study_id=phs003510.v1.p1) |
| BioLINCC_HFN-INDIE | Heart Failure Network: Inorganic Nitrite Delivery to Improve Exercise Capacity in HFpEF (HFN INDIE-BioLINCC) | Heart Failure | Clinical Trial | 105 | 294 | [phs003667.v1.p1.c1](https://www.ncbi.nlm.nih.gov/projects/gap/cgi-bin/study.cgi?study_id=phs003667.v1.p1) |
| BIOLINCC_HFN-RELAX | Heart Failure Network - Phosphodiesterase-5 Inhibition to Improve Clinical Status and Exercise Capacity in Diastolic Heart Failure (HFN RELAX-BioLINCC) |  | Clinical Trial | 216 | 161 | [phs003565.v1.p1.c1](https://www.ncbi.nlm.nih.gov/projects/gap/cgi-bin/study.cgi?study_id=phs003565.v1.p1) |
| BioLINCC_HFN-ROSE | Heart Failure Network - Renal Optimization Strategies Evaluation in Acute Heart Failure and Reliable Evaluation of Dyspnea (HFN ROSE-BioLINCC) |  | Clinical Trial | 360 | 233 | [phs003589.v1.p1.c1](https://www.ncbi.nlm.nih.gov/projects/gap/cgi-bin/study.cgi?study_id=phs003589.v1.p1) |
| BioLINCC_HHP-BioLINCC | Honolulu Heart Program |  | Prospective Longitudinal Cohort | 8006 | 2555 | [phs003907.v1.p1.c1](https://www.ncbi.nlm.nih.gov/projects/gap/cgi-bin/study.cgi?study_id=phs003907.v1.p1) |
| BioLINCC_JHS | Jackson Heart Study (JHS) BioLINCC |  | Prospective Longitudinal Cohort | 711 | 2732 | [phs003740.v1.p1.c2](https://www.ncbi.nlm.nih.gov/projects/gap/cgi-bin/study.cgi?study_id=phs003740.v1.p1) |
| BioLINCC_JHS | Jackson Heart Study (JHS) BioLINCC |  | Prospective Longitudinal Cohort | 2567 | 2732 | [phs003740.v1.p1.c1](https://www.ncbi.nlm.nih.gov/projects/gap/cgi-bin/study.cgi?study_id=phs003740.v1.p1) |
| BioLINCC_JHS | Jackson Heart Study (JHS) BioLINCC |  | Prospective Longitudinal Cohort | 201 | 2732 | [phs003740.v1.p1.c4](https://www.ncbi.nlm.nih.gov/projects/gap/cgi-bin/study.cgi?study_id=phs003740.v1.p1) |
| BioLINCC_JHS | Jackson Heart Study (JHS) BioLINCC |  | Prospective Longitudinal Cohort | 404 | 2732 | [phs003740.v1.p1.c3](https://www.ncbi.nlm.nih.gov/projects/gap/cgi-bin/study.cgi?study_id=phs003740.v1.p1) |
| BioLINCC_LTRC-BioLINCC | Lung Tissue Research Consortium |  | Cross-Sectional | 4486 | 3 | [phs003913.v1.p1.c1](https://www.ncbi.nlm.nih.gov/projects/gap/cgi-bin/study.cgi?study_id=phs003913.v1.p1) |
| BioLINCC_MESA | Multi-Ethnic Study of Atherosclerosis (BioLINCC) |  | Prospective Longitudinal Cohort | 6043 | 2624 | [phs003288.v1.p1.c1](https://www.ncbi.nlm.nih.gov/projects/gap/cgi-bin/study.cgi?study_id=phs003288.v1.p1) |
| BioLINCC_MESA | Multi-Ethnic Study of Atherosclerosis (BioLINCC) |  | Prospective Longitudinal Cohort | 771 | 2624 | [phs003288.v1.p1.c2](https://www.ncbi.nlm.nih.gov/projects/gap/cgi-bin/study.cgi?study_id=phs003288.v1.p1) |
| BioLINCC_PAD-BioLINCC | Public Access Defibrillation Community Trial (PAD)(PAD-BioLINCC) |  | Clinical Trial | 3951 | 374 | [phs003858.v1.p1.c1](https://www.ncbi.nlm.nih.gov/projects/gap/cgi-bin/study.cgi?study_id=phs003858.v1.p1) |
| BioLINCC_PETAL_BLUE_CORAL_v2 | BLUE CORAL: Biology and Longitudinal Epidemiology of PETAL COVID-19 Observational Study |  | Clinical Trial | 1376 | 538 | [phs003419.v2.p1.c1](https://www.ncbi.nlm.nih.gov/projects/gap/cgi-bin/study.cgi?study_id=phs003419.v1.p1) |
| BioLINCC_PETAL_ROSE | Prevention and Early Treatment of Acute Lung Injury Network – Reevaluation of Systemic Early Neuromuscular Blockade (PETAL ROSE) |  | Clinical Trial | 1006 | 313 | [phs003878.v1.p1.c1](https://www.ncbi.nlm.nih.gov/projects/gap/cgi-bin/study.cgi?study_id=phs003878.v1.p1) |
| BioLINCC_PETAL_VIOLET | Prevention and Early Treatment of Acute Lung Injury Network – Vitamin D to Improve Outcomes by Leveraging Early Treatment (PETAL VIOLET) |  | Clinical Trial | 1358 | 221 | [phs003879.v1.p1.c1](https://www.ncbi.nlm.nih.gov/projects/gap/cgi-bin/study.cgi?study_id=phs003879.v1.p1) |
| BioLINCC_PETAL-ASTER | Prevention and Early Treatment of Acute Lung Injury (PETAL) Acetaminophen in Sepsis: Targeted Therapy to Enhance Recovery |  | Clinical Trial | 487 | 238 | [phs003900.v1.p1.c1](https://www.ncbi.nlm.nih.gov/projects/gap/cgi-bin/study.cgi?study_id=phs003900.v1.p1) |
| BioLINCC_PETAL-LOTUS-FRUIT | Prevention and Early Treatment of Acute Lung Injury - Low Tidal Volume Universal (PETAL-LOTUS FRUIT) |  | Prospective Longitudinal Cohort | 2848 | 81 | [phs003791.v1.p1.c1](https://www.ncbi.nlm.nih.gov/projects/gap/cgi-bin/study.cgi?study_id=phs003791.v1.p1) |
| BioLINCC_PF-ILD | Proteomic Biomarkers of Progressive Fibrosing Interstitial Lung Disease: a Multicentre Cohort Analysis (PF-ILD Proteomics-BioLINCC) |  | Prospective Longitudinal Cohort | 589 | 382 | [phs003954.v1.p1.c1](https://www.ncbi.nlm.nih.gov/projects/gap/cgi-bin/study.cgi?study_id=phs003954.v1.p1) |
| BioLINCC_REACT | Rapid Early Action for Coronary Treatment (REACT) |  | Clinical Trial | 92404 | 213 | [phs003885.v1.p1.c1](https://www.ncbi.nlm.nih.gov/projects/gap/cgi-bin/study.cgi?study_id=phs003885.v1.p1) |
| BioLINCC_RESTORE | Randomized Evaluation of Sedation Titration for Respiratory Failure (RESTORE-BioLINCC) |  | Clinical Trial | 2449 | 359 | [phs003783.v1.p1.c1](https://www.ncbi.nlm.nih.gov/projects/gap/cgi-bin/study.cgi?study_id=phs003783.v1.p1) |
| BioLINCC_ROC_ALPS | Resuscitation Outcomes Consortium (ROC) Amiodarone, Lidocaine or Neither for Out-Of-Hospital Cardiac Arrest Due to Ventricular Fibrillation or Ventricular Tachycardia (ALPS) |  | Clinical Trial | 4653 | 614 | [phs003784.v1.p1.c1](https://www.ncbi.nlm.nih.gov/projects/gap/cgi-bin/study.cgi?study_id=phs003784.v1.p1) |
| BioLINCC_ROC_Cardiac_Epistry_1_2 | Resuscitation Outcomes Consortium (ROC) Cardiac Epidemiologic Registry (Cardiac Epistry) Versions 1 and 2 (ROC-Cardiac Epistry 1 and 2-BioLINCC) |  | Prospective Longitudinal Cohort | 109326 | 278 | [phs003803.v1.p1.c1](https://www.ncbi.nlm.nih.gov/projects/gap/cgi-bin/study.cgi?study_id=phs003803.v1.p1) |
| BioLINCC_ROC_PROHS | Resuscitation Outcomes Consortium (ROC) Prehospital Resuscitation on Helicopter Study (PROHS)(ROC-PROHS-BioLINCC) |  | Prospective Longitudinal Cohort | 2341 | 99 | [phs003826.v1.p1.c1](https://www.ncbi.nlm.nih.gov/projects/gap/cgi-bin/study.cgi?study_id=phs003826.v1.p1) |
| BioLINCC_ROC-CCC | Resuscitation Outcomes Consortium Trial of Continuous Compressions Versus Standard CPR in Patients With out-of-Hospital Cardiac Arrest (ROC CCC-BioLINCC) |  | Clinical Trial | 26148 | 497 | [phs003901.v1.p1.c1](https://www.ncbi.nlm.nih.gov/projects/gap/cgi-bin/study.cgi?study_id=phs003901.v1.p1) |
| BioLINCC_ROC-HS-TBI | Resuscitation Outcomes Consortium (ROC) Hypertonic Saline (HS) Trial Shock Study and Traumatic Brain Injury Study (TBI) (ROC-HS/TBI-BioLINCC) |  | Clinical Trial | 2220 | 827 | [phs003777.v1.p1.c1](https://www.ncbi.nlm.nih.gov/projects/gap/cgi-bin/study.cgi?study_id=phs003777.v1.p1) |
| BioLINCC_ROC-PART | Resuscitation Outcomes Consortium Pragmatic Trial of Airway Management in out-of-Hospital Cardiac Arrest (ROC PART-BioLINCC) |  | Clinical Trial | 3004 | 404 | [phs003902.v1.p1.c1](https://www.ncbi.nlm.nih.gov/projects/gap/cgi-bin/study.cgi?study_id=phs003902.v1.p1) |
| BioLINCC_ROC-Trauma_Epistry | Resuscitation Outcomes Consortium (ROC) Trauma Epidemiologic Registry (Trauma Epistry) (ROC-Trauma Epistry-BioLINCC) |  | Prospective Longitudinal Cohort | 13730 | 179 | [phs003809.v1.p1.c1](https://www.ncbi.nlm.nih.gov/projects/gap/cgi-bin/study.cgi?study_id=phs003809.v1.p1) |
| BioLINCC_SHHS | Sleep Heart Health Study (SHHS-BioLINCC) | Sleep Apnea, Obstructive | Prospective Longitudinal Cohort | 5839 | 655 | [phs003637.v1.p1.c1](https://www.ncbi.nlm.nih.gov/projects/gap/cgi-bin/study.cgi?study_id=phs003637.v1.p1) |
| BioLINCC_SOLVD | Studies of Left Ventricular Dysfunction (SOLVD-BioLINCC) | Cardiovascular Diseases | Clinical Trial | 11939 | 553 | [phs003668.v1.p1.c1](https://www.ncbi.nlm.nih.gov/projects/gap/cgi-bin/study.cgi?study_id=phs003668.v1.p1) |
| BioLINCC_SPRINT | Systolic Blood Pressure Intervention Trial (SPRINT-BioLINCC) | Hypertension | Clinical Trial | 9361 | 2748 | [phs003483.v1.p1.c1](https://www.ncbi.nlm.nih.gov/projects/gap/cgi-bin/study.cgi?study_id=phs003483.v1.p1) |
| BioLINCC_TOLSURF | Trial of Late Surfactant for Prevention of Bronchopulmonary Dysplasia: A Study in Ventilated Preterm Infants Receiving Inhaled Nitric Oxide |  | Clinical Trial | 511 | 1128 | [phs003899.v1.p1.c1](https://www.ncbi.nlm.nih.gov/projects/gap/cgi-bin/study.cgi?study_id=phs003899.v1.p1) |
| BioLINCC_TOPCAT | Treatment of Preserved Cardiac Function Heart Failure with an Aldosterone Antagonist (TOPCAT-BioLINCC) |  | Clinical Trial | 3445 | 4 | [phs003665.v1.p1.c1](https://www.ncbi.nlm.nih.gov/projects/gap/cgi-bin/study.cgi?study_id=phs003665.v1.p1) |
| BIOME | NHLBI TOPMed - NHGRI CCDG: The BioMe Biobank at Mount Sinai | Coronary Artery Disease | Prospective Longitudinal Cohort | 12050 | 32 | [phs001644.v3.p2.c1](https://www.ncbi.nlm.nih.gov/projects/gap/cgi-bin/study.cgi?study_id=phs001644.v3.p2) |
| BioVU_AF | NHLBI TOPMed - NHGRI CCDG: The Vanderbilt University BioVU Atrial Fibrillation Genetics Study | Atrial Fibrillation | Case-Control | 2666 | 26 | [phs001624.v3.p2.c1](https://www.ncbi.nlm.nih.gov/projects/gap/cgi-bin/study.cgi?study_id=phs001624.v3.p2) |
| BL_ACCORD | Action to Control Cardiovascular Risk in Diabetes (ACCORD-BioLINCC) | Diabetes Mellitus | Clinical Trial | 10251 | 186 | [phs003551.v1.p1.c1](https://www.ncbi.nlm.nih.gov/projects/gap/cgi-bin/study.cgi?study_id=phs003551.v1.p1) |
| BL_HFN_IRONOUT | Oral Iron Repletion Effects on Oxygen Uptake in Heart Failure (IRONOUT) | Heart Failure | Clinical Trial | 225 | 179 | [phs003557.v1.p1.c1](https://www.ncbi.nlm.nih.gov/projects/gap/cgi-bin/study.cgi?study_id=phs003557.v1.p1) |
| BL_HFN-FIGHT | Heart Failure Network: Functional Impact of GLP-1 for Heart Failure Treatment (HFN FIGHT-BioLINCC) | Heart Failure | Clinical Trial | 300 | 250 | [phs003542.v1.p1.c1](https://www.ncbi.nlm.nih.gov/projects/gap/cgi-bin/study.cgi?study_id=phs003542.v1.p1) |
| BMT_CTN-0601 | Unrelated Donor Reduced Intensity Bone Marrow Transplant for Children with Severe Sickle Cell Disease (BMT CTN-0601-BioLINCC) | Anemia, Hemolytic, Congenital | Clinical Trial | 55 | 273 | [phs003470.v1.p1.c1](https://www.ncbi.nlm.nih.gov/projects/gap/cgi-bin/study.cgi?study_id=phs003470.v1.p1) |
| C3PO | Clinical-trial of COVID-19 Convalescent Plasma in Outpatients | COVID-19 | Clinical Trial | 511 | 159 | [phs002752.v2.p1.c1](https://www.ncbi.nlm.nih.gov/projects/gap/cgi-bin/study.cgi?study_id=phs002752.v1.p1) |
| C4R_JHS | The Collaborative Cohort of Cohorts for COVID-19 Research (C4R) |  | Cohort | 201 | 247 | [phs002907.v1.p1.c2](https://www.ncbi.nlm.nih.gov/projects/gap/cgi-bin/study.cgi?study_id=phs002907.v1.p1) |
| C4R_JHS | The Collaborative Cohort of Cohorts for COVID-19 Research (C4R) |  | Cohort | 878 | 247 | [phs002907.v1.p1.c1](https://www.ncbi.nlm.nih.gov/projects/gap/cgi-bin/study.cgi?study_id=phs002907.v1.p1) |
| C4R_JHS | The Collaborative Cohort of Cohorts for COVID-19 Research (C4R) |  | Cohort | 2289 | 247 | [phs002907.v1.p1.c3](https://www.ncbi.nlm.nih.gov/projects/gap/cgi-bin/study.cgi?study_id=phs002907.v1.p1) |
| C4R_JHS | The Collaborative Cohort of Cohorts for COVID-19 Research (C4R) |  | Cohort | 515 | 247 | [phs002907.v1.p1.c4](https://www.ncbi.nlm.nih.gov/projects/gap/cgi-bin/study.cgi?study_id=phs002907.v1.p1) |
| C4R_SPIROMICS | Collaborative Cohort of Cohorts for COVID-19 Research (C4R): Subpopulations and Intermediate Outcome Measures in COPD Study (SPIROMICS) | COVID-19 | Prospective Longitudinal Cohort | 1191 | 280 | [phs002909.v1.p1.c1](https://www.ncbi.nlm.nih.gov/projects/gap/cgi-bin/study.cgi?study_id=phs002909.v1.p1) |
| C4R_SPIROMICS | Collaborative Cohort of Cohorts for COVID-19 Research (C4R): Subpopulations and Intermediate Outcome Measures in COPD Study (SPIROMICS) | COVID-19 | Prospective Longitudinal Cohort | 26 | 280 | [phs002909.v1.p1.c6](https://www.ncbi.nlm.nih.gov/projects/gap/cgi-bin/study.cgi?study_id=phs002909.v1.p1) |
| C4R_SPIROMICS | Collaborative Cohort of Cohorts for COVID-19 Research (C4R): Subpopulations and Intermediate Outcome Measures in COPD Study (SPIROMICS) | COVID-19 | Prospective Longitudinal Cohort | 7 | 280 | [phs002909.v1.p1.c5](https://www.ncbi.nlm.nih.gov/projects/gap/cgi-bin/study.cgi?study_id=phs002909.v1.p1) |
| C4R_SPIROMICS | Collaborative Cohort of Cohorts for COVID-19 Research (C4R): Subpopulations and Intermediate Outcome Measures in COPD Study (SPIROMICS) | COVID-19 | Prospective Longitudinal Cohort | 6 | 280 | [phs002909.v1.p1.c7](https://www.ncbi.nlm.nih.gov/projects/gap/cgi-bin/study.cgi?study_id=phs002909.v1.p1) |
| C4R_SPIROMICS | Collaborative Cohort of Cohorts for COVID-19 Research (C4R): Subpopulations and Intermediate Outcome Measures in COPD Study (SPIROMICS) | COVID-19 | Prospective Longitudinal Cohort | 17 | 280 | [phs002909.v1.p1.c8](https://www.ncbi.nlm.nih.gov/projects/gap/cgi-bin/study.cgi?study_id=phs002909.v1.p1) |
| C4R_SPIROMICS | Collaborative Cohort of Cohorts for COVID-19 Research (C4R): Subpopulations and Intermediate Outcome Measures in COPD Study (SPIROMICS) | COVID-19 | Prospective Longitudinal Cohort | 40 | 280 | [phs002909.v1.p1.c4](https://www.ncbi.nlm.nih.gov/projects/gap/cgi-bin/study.cgi?study_id=phs002909.v1.p1) |
| C4R_SPIROMICS | Collaborative Cohort of Cohorts for COVID-19 Research (C4R): Subpopulations and Intermediate Outcome Measures in COPD Study (SPIROMICS) | COVID-19 | Prospective Longitudinal Cohort | 197 | 280 | [phs002909.v1.p1.c2](https://www.ncbi.nlm.nih.gov/projects/gap/cgi-bin/study.cgi?study_id=phs002909.v1.p1) |
| C4R_SPIROMICS | Collaborative Cohort of Cohorts for COVID-19 Research (C4R): Subpopulations and Intermediate Outcome Measures in COPD Study (SPIROMICS) | COVID-19 | Prospective Longitudinal Cohort | 95 | 280 | [phs002909.v1.p1.c3](https://www.ncbi.nlm.nih.gov/projects/gap/cgi-bin/study.cgi?study_id=phs002909.v1.p1) |
| CAMP | NHLBI TOPMed: Childhood Asthma Management Program (CAMP) | Asthma | Parent-Offspring Trios | 2785 | 74 | [phs001726.v3.p1.c1](https://www.ncbi.nlm.nih.gov/projects/gap/cgi-bin/study.cgi?study_id=phs001726.v3.p1) |
| CardioATVB | Center for Common Disease Genomics [CCDG] - Cardiovascular ATVB: Atherosclerosis Thrombosis and Vascular Biology | Atherosclerosis | Case-Control | 58 | 9 | [phs001592.v1.p1.c1](https://www.ncbi.nlm.nih.gov/projects/gap/cgi-bin/study.cgi?study_id=phs001592.v1.p1) |
| CARE_BADGER | NHLBI TOPMed: Best ADd-on Therapy Giving Effective Response (BADGER) | Asthma | Prospective Longitudinal Cohort | 50 | 24 | [phs001728.v3.p1.c2](https://www.ncbi.nlm.nih.gov/projects/gap/cgi-bin/study.cgi?study_id=phs001728.v3.p1) |
| CARE_CLIC | NHLBI TOPMed: Characterizing the Response to a Leukotriene Receptor Antagonist and an Inhaled Corticosteroid (CLIC) | Asthma | Prospective Longitudinal Cohort | 19 | 24 | [phs001729.v3.p1.c2](https://www.ncbi.nlm.nih.gov/projects/gap/cgi-bin/study.cgi?study_id=phs001729.v3.p1) |
| CARE_PACT | NHLBI TOPMed: Pediatric Asthma Controller Trial (PACT) | Asthma | Prospective Longitudinal Cohort | 41 | 24 | [phs001730.v2.p1.c2](https://www.ncbi.nlm.nih.gov/projects/gap/cgi-bin/study.cgi?study_id=phs001730.v2.p1) |
| CARE_TREXA | NHLBI TOPMed: TReating Children to Prevent EXacerbations of Asthma (TREXA) | Asthma | Prospective Longitudinal Cohort | 89 | 24 | [phs001732.v2.p1.c2](https://www.ncbi.nlm.nih.gov/projects/gap/cgi-bin/study.cgi?study_id=phs001732.v2.p1) |
| CATHGEN | CATHeterization GENetics (CATHGEN) | Coronary Disease | Cross-Sectional (clinical) and Case Set (genomic) | 3304 | 7 | [phs000703.v1.p1.c1](https://www.ncbi.nlm.nih.gov/projects/gap/cgi-bin/study.cgi?study_id=phs000703.v1.p1) |
| CATHGEN | NHLBI TOPMed: NHGRI CCDG: Early-onset Atrial Fibrillation in the CATHeterization GENetics (CATHGEN) Cohort | Atrial Fibrillation | Cross-Sectional (clinical) and Case Set (genomic) | 1271 | 24 | [phs001600.v2.p2.c1](https://www.ncbi.nlm.nih.gov/projects/gap/cgi-bin/study.cgi?study_id=phs001600.v2.p2) |
| CCAF | NHLBI TOPMed: Cleveland Clinic Atrial Fibrillation (CCAF) Study | Atrial Fibrillation | Case Set | 363 | 37 | [phs001189.v5.p1.c1](https://www.ncbi.nlm.nih.gov/projects/gap/cgi-bin/study.cgi?study_id=phs001189.v5.p1) |
| CCAF | The Cleveland Clinic Foundation's Lone Atrial Fibrillation GWAS Study | Atrial Fibrillation | Case Set | 543 | 14 | [phs000820.v1.p1.c1](https://www.ncbi.nlm.nih.gov/projects/gap/cgi-bin/study.cgi?study_id=phs000820.v1.p1) |
| CCDG_PMBB | NHLBI TOPMed - NHGRI CCDG: Penn Medicine BioBank Early Onset Atrial Fibrillation Study | Atrial Fibrillation | Case Set | 2210 | 25 | [phs001601.v2.p2.c1](https://www.ncbi.nlm.nih.gov/projects/gap/cgi-bin/study.cgi?study_id=phs001601.v2.p2) |
| CFS | NHLBI Cleveland Family Study (CFS) Candidate Gene Association Resource (CARe) | Sleep Apnea Syndromes | Prospective Longitudinal Cohort | 1473 | 2320 | [phs000284.v2.p1.c1](https://www.ncbi.nlm.nih.gov/projects/gap/cgi-bin/study.cgi?study_id=phs000284.v2.p1) |
| CFS | NHLBI TOPMed: The Cleveland Family Study (CFS) | Sleep Apnea Syndromes | Prospective Longitudinal Cohort | 1293 | 14 | [phs000954.v4.p2.c1](https://www.ncbi.nlm.nih.gov/projects/gap/cgi-bin/study.cgi?study_id=phs000954.v4.p2) |
| ChildrensHS_GAP | NHLBI TOPMed: Children's Health Study (CHS) Integrative Genetic Approaches to Gene-Air Pollution Interactions in Asthma (GAP) | Asthma | Case-Control | 7 | 24 | [phs001602.v2.p1.c1](https://www.ncbi.nlm.nih.gov/projects/gap/cgi-bin/study.cgi?study_id=phs001602.v2.p1) |
| ChildrensHS_IGERA | NHLBI TOPMed: Children's Health Study (CHS) Integrative Genomics and Environmental Research of Asthma (IGERA) | Asthma | Case-Control | 160 | 25 | [phs001603.v2.p1.c1](https://www.ncbi.nlm.nih.gov/projects/gap/cgi-bin/study.cgi?study_id=phs001603.v2.p1) |
| ChildrensHS_MetaAir | NHLBI TOPMed: Children's Health Study (CHS) Effects of Air Pollution on the Development of Obesity in Children (Meta-AIR) | Asthma | Case-Control | 56 | 25 | [phs001604.v2.p1.c1](https://www.ncbi.nlm.nih.gov/projects/gap/cgi-bin/study.cgi?study_id=phs001604.v2.p1) |
| CHIRAH | NHLBI TOPMed: Chicago Initiative to Raise Asthma Health Equity (CHIRAH) | Asthma | Case Set | 292 | 25 | [phs001605.v2.p1.c2](https://www.ncbi.nlm.nih.gov/projects/gap/cgi-bin/study.cgi?study_id=phs001605.v2.p1) |
| CHS | Cardiovascular Health Study (CHS) Cohort: an NHLBI-funded observational study of risk factors for cardiovascular disease in adults 65 years or older | Cardiovascular Diseases | Prospective Longitudinal Cohort | 8 | 14551 | [phs000287.v7.p1.c4](https://www.ncbi.nlm.nih.gov/projects/gap/cgi-bin/study.cgi?study_id=phs000287.v7.p1) |
| CHS | Cardiovascular Health Study (CHS) Cohort: an NHLBI-funded observational study of risk factors for cardiovascular disease in adults 65 years or older | Cardiovascular Diseases | Prospective Longitudinal Cohort | 217 | 14551 | [phs000287.v7.p1.c2](https://www.ncbi.nlm.nih.gov/projects/gap/cgi-bin/study.cgi?study_id=phs000287.v7.p1) |
| CHS | Cardiovascular Health Study (CHS) Cohort: an NHLBI-funded observational study of risk factors for cardiovascular disease in adults 65 years or older | Cardiovascular Diseases | Prospective Longitudinal Cohort | 2 | 14551 | [phs000287.v7.p1.c3](https://www.ncbi.nlm.nih.gov/projects/gap/cgi-bin/study.cgi?study_id=phs000287.v7.p1) |
| CHS | Cardiovascular Health Study (CHS) Cohort: an NHLBI-funded observational study of risk factors for cardiovascular disease in adults 65 years or older | Cardiovascular Diseases | Prospective Longitudinal Cohort | 5382 | 14551 | [phs000287.v7.p1.c1](https://www.ncbi.nlm.nih.gov/projects/gap/cgi-bin/study.cgi?study_id=phs000287.v7.p1) |
| CHS | NHLBI TOPMed: Cardiovascular Health Study | Cardiovascular Diseases | Prospective Longitudinal Cohort | 3 | 17 | [phs001368.v4.p2.c4](https://www.ncbi.nlm.nih.gov/projects/gap/cgi-bin/study.cgi?study_id=phs001368.v4.p2) |
| CHS | NHLBI TOPMed: Cardiovascular Health Study | Cardiovascular Diseases | Prospective Longitudinal Cohort | 4743 | 17 | [phs001368.v4.p2.c1](https://www.ncbi.nlm.nih.gov/projects/gap/cgi-bin/study.cgi?study_id=phs001368.v4.p2) |
| CHS | NHLBI TOPMed: Cardiovascular Health Study | Cardiovascular Diseases | Prospective Longitudinal Cohort | 1 | 17 | [phs001368.v4.p2.c3](https://www.ncbi.nlm.nih.gov/projects/gap/cgi-bin/study.cgi?study_id=phs001368.v4.p2) |
| CHS | NHLBI TOPMed: Cardiovascular Health Study | Cardiovascular Diseases | Case-Control | 130 | 17 | [phs001368.v4.p2.c2](https://www.ncbi.nlm.nih.gov/projects/gap/cgi-bin/study.cgi?study_id=phs001368.v4.p2) |
| COPDGENE | Genetic Epidemiology of COPD (COPDGene) Funded by the National Heart, Lung, and Blood Institute | Pulmonary Disease, Chronic Obstructive | Case-Control | 10099 | 337 | [phs000179.v6.p2.c1](https://www.ncbi.nlm.nih.gov/projects/gap/cgi-bin/study.cgi?study_id=phs000179.v6.p2) |
| COPDGENE | Genetic Epidemiology of COPD (COPDGene) Funded by the National Heart, Lung, and Blood Institute | Pulmonary Disease, Chronic Obstructive | Case-Control | 272 | 337 | [phs000179.v6.p2.c2](https://www.ncbi.nlm.nih.gov/projects/gap/cgi-bin/study.cgi?study_id=phs000179.v6.p2) |
| COPDGENE | Genetic Epidemiology of COPD Study (COPDGene) - The Collaborative Cohort of Cohorts for COVID-19 Research (C4R) | COVID-19 | Case-Control | 116 | 378 | [phs002910.v1.p1.c2](https://www.ncbi.nlm.nih.gov/projects/gap/cgi-bin/study.cgi?study_id=phs002910.v1.p1) |
| COPDGENE | Genetic Epidemiology of COPD Study (COPDGene) - The Collaborative Cohort of Cohorts for COVID-19 Research (C4R) | COVID-19 | Case-Control | 4075 | 378 | [phs002910.v1.p1.c1](https://www.ncbi.nlm.nih.gov/projects/gap/cgi-bin/study.cgi?study_id=phs002910.v1.p1) |
| COPDGENE | NHLBI TOPMed: Genetic Epidemiology of COPD (COPDGene) in the TOPMed Program | Pulmonary Disease, Chronic Obstructive | Case-Control | 10367 | 68 | [phs000951.v6.p5.c1](https://www.ncbi.nlm.nih.gov/projects/gap/cgi-bin/study.cgi?study_id=phs000951.v6.p5) |
| COPDGENE | NHLBI TOPMed: Genetic Epidemiology of COPD (COPDGene) in the TOPMed Program | Pulmonary Disease, Chronic Obstructive | Case-Control | 293 | 68 | [phs000951.v6.p5.c2](https://www.ncbi.nlm.nih.gov/projects/gap/cgi-bin/study.cgi?study_id=phs000951.v6.p5) |
| CRA | NHLBI TOPMed: The Genetic Epidemiology of Asthma in Costa Rica | Asthma | Family/Twin/Trios | 4283 | 78 | [phs000988.v6.p1.c1](https://www.ncbi.nlm.nih.gov/projects/gap/cgi-bin/study.cgi?study_id=phs000988.v6.p1) |
| CSSCD | Cooperative Study of Sickle Cell Disease (CSSCD) | Anemia, Sickle Cell | Clinical Trial | 4085 | 7519 | [phs002362.v1.p1.c1](https://www.ncbi.nlm.nih.gov/projects/gap/cgi-bin/study.cgi?study_id=phs002362.v1.p1) |
| DECAF | NHLBI TOPMed: Determining the Association of Chromosomal Variants with Non-PV Triggers and Ablation-Outcome in AF (DECAF) | Atrial Fibrillation | Case Set | 6 | 19 | [phs001546.v2.p1.c1](https://www.ncbi.nlm.nih.gov/projects/gap/cgi-bin/study.cgi?study_id=phs001546.v2.p1) |
| DHS | The Diabetes Heart Study (DHS) | Cardiovascular Diseases | Cross-Sectional | 1177 | 28 | [phs001012.v1.p1.c1](https://www.ncbi.nlm.nih.gov/projects/gap/cgi-bin/study.cgi?study_id=phs001012.v1.p1) |
| DIR | A Pilot Trial of Complement Inhibition Using Eculizumab to Overcome Platelet Transfusion Refractoriness in HLA Allo-Immunized Patients | Thrombocytopenia | Clinical Trial | 10 | 21 | [phs003212.v1.p1.c1](https://www.ncbi.nlm.nih.gov/projects/gap/cgi-bin/study.cgi?study_id=phs003212.v1.p1) |
| DIR_ApoA1 | ApoA-1 and Atherosclerosis in Psoriasis | Atherosclerosis | Prospective Longitudinal Cohort | 310 | 46 | [phs003231.v1.p1.c1](https://www.ncbi.nlm.nih.gov/projects/gap/cgi-bin/study.cgi?study_id=phs003231.v1.p1) |
| ECLIPSE | Evaluation of COPD Longitudinally to Identify Predictive Surrogate Endpoints (ECLIPSE) | Pulmonary Disease, Chronic Obstructive | Case-Control | 2746 | 808 | [phs001252.v1.p1.c1](https://www.ncbi.nlm.nih.gov/projects/gap/cgi-bin/study.cgi?study_id=phs001252.v1.p1) |
| ECLIPSE | NHLBI TOPMed: Evaluation of COPD Longitudinally to Identify Predictive Surrogate Endpoints (ECLIPSE) | Pulmonary Disease, Chronic Obstructive | Case-Control | 2331 | 14 | [phs001472.v3.p2.c1](https://www.ncbi.nlm.nih.gov/projects/gap/cgi-bin/study.cgi?study_id=phs001472.v3.p2) |
| EGCUT | NHLBI TOPMed: Early-Onset Atrial Fibrillation in the Estonian Biobank | Atrial Fibrillation | Case Set | 324 | 18 | [phs001606.v3.p1.c1](https://www.ncbi.nlm.nih.gov/projects/gap/cgi-bin/study.cgi?study_id=phs001606.v3.p1) |
| EOCOPD | NHLBI TOPMed: Boston Early-Onset COPD Study | Pulmonary Disease, Chronic Obstructive | Family/Twin/Trios | 74 | 14 | [phs000946.v6.p2.c1](https://www.ncbi.nlm.nih.gov/projects/gap/cgi-bin/study.cgi?study_id=phs000946.v6.p2) |
| Exome_SCID | National Heart Lung and Blood Institute Exome sequencing in SCID | Severe Combined Immunodeficiency | Parent-Offspring Trios | 3 | 36 | [phs000479.v1.p1.c1](https://www.ncbi.nlm.nih.gov/projects/gap/cgi-bin/study.cgi?study_id=phs000479.v1.p1) |
| exRNA_CSF | Profiles of Extracellular RNA in Cerebrospinal Fluid and Plasma from Subarachnoid Hemorrhage Patients | Circulating MicroRNA | Case Set | 7 | 23 | [phs001759.v1.p1.c1](https://www.ncbi.nlm.nih.gov/projects/gap/cgi-bin/study.cgi?study_id=phs001759.v1.p1) |
| Fam_MLD | NHLBI GO-ESP: Family Studies (Mendelian Lipid Disorders) | Hyperlipoproteinemia Type II | Case Set | 29 | 14 | [phs000587.v1.p1.c1](https://www.ncbi.nlm.nih.gov/projects/gap/cgi-bin/study.cgi?study_id=phs000587.v1.p1) |
| FamExome_RarePeds | Familial Exome Sequencing in Rare Pediatric Phenotypes | Mendelian Randomization Analysis | Prospective Longitudinal Cohort | 6 | 30 | [phs000553.v1.p1.c1](https://www.ncbi.nlm.nih.gov/projects/gap/cgi-bin/study.cgi?study_id=phs000553.v1.p1) |
| FHS | Framingham Cohort | Cardiovascular Diseases | Prospective Longitudinal Cohort | 13070 | 62547 | [phs000007.v31.p12.c1](https://www.ncbi.nlm.nih.gov/projects/gap/cgi-bin/study.cgi?study_id=phs000007.v31.p12) |
| FHS | Framingham Cohort | Cardiovascular Diseases | Prospective Longitudinal Cohort | 2079 | 62547 | [phs000007.v31.p12.c2](https://www.ncbi.nlm.nih.gov/projects/gap/cgi-bin/study.cgi?study_id=phs000007.v31.p12) |
| FHS | Framingham Heart Study (FHS) - The Collaborative Cohort of Cohorts for COVID-19 Research (C4R) | COVID-19 | Prospective Longitudinal Cohort | 847 | 498 | [phs002911.v1.p1.c2](https://www.ncbi.nlm.nih.gov/projects/gap/cgi-bin/study.cgi?study_id=phs002911.v1.p1) |
| FHS | Framingham Heart Study (FHS) - The Collaborative Cohort of Cohorts for COVID-19 Research (C4R) | COVID-19 | Prospective Longitudinal Cohort | 6423 | 498 | [phs002911.v1.p1.c1](https://www.ncbi.nlm.nih.gov/projects/gap/cgi-bin/study.cgi?study_id=phs002911.v1.p1) |
| FHS | NHLBI TOPMed: Genomic Activities such as Whole Genome Sequencing and Related Phenotypes in the Framingham Heart Study | Cardiovascular Diseases | Prospective Longitudinal Cohort | 6396 | 1399 | [phs000974.v6.p5.c1](https://www.ncbi.nlm.nih.gov/projects/gap/cgi-bin/study.cgi?study_id=phs000974.v6.p5) |
| FHS | NHLBI TOPMed: Genomic Activities such as Whole Genome Sequencing and Related Phenotypes in the Framingham Heart Study | Cardiovascular Diseases | Prospective Longitudinal Cohort | 908 | 1399 | [phs000974.v6.p5.c2](https://www.ncbi.nlm.nih.gov/projects/gap/cgi-bin/study.cgi?study_id=phs000974.v6.p5) |
| GALA | NHLBI TOPMed: Genetics of Asthma in Latino Americans (GALA) | Asthma | Case Set | 1024 | 26 | [phs001542.v2.p1.c2](https://www.ncbi.nlm.nih.gov/projects/gap/cgi-bin/study.cgi?study_id=phs001542.v2.p1) |
| GALAII | Genes-Environments and Admixture in Latino Asthmatics (GALA II) Study | Lung Diseases | Case-Control (clinical) and case set (genomic) | 4458 | 26 | [phs001180.v2.p1.c2](https://www.ncbi.nlm.nih.gov/projects/gap/cgi-bin/study.cgi?study_id=phs001180.v2.p1) |
| GALAII | NHLBI TOPMed: Genes-environments and Admixture in Latino Asthmatics (GALA II) Study | Lung Diseases | Case-Control (clinical) and case set (genomic) | 4860 | 24 | [phs000920.v6.p4.c2](https://www.ncbi.nlm.nih.gov/projects/gap/cgi-bin/study.cgi?study_id=phs000920.v6.p4) |
| GCPD-A | NHLBI TOPMed: Genetic Causes of Complex Pediatric Disorders - Asthma (GCPD-A) | Asthma | Case-Control | 5464 | 26 | [phs001661.v3.p1.c1](https://www.ncbi.nlm.nih.gov/projects/gap/cgi-bin/study.cgi?study_id=phs001661.v3.p1) |
| GENAF | NHLBI TOPMed - NHGRI CCDG: The GENetics in Atrial Fibrillation (GENAF) Study | Atrial Fibrillation | Case Set | 90 | 18 | [phs001547.v3.p1.c1](https://www.ncbi.nlm.nih.gov/projects/gap/cgi-bin/study.cgi?study_id=phs001547.v3.p1) |
| GENESTAR | GeneSTAR (Genetic Study of Atherosclerosis Risk) NextGen Consortium: Functional Genomics of Platelet Aggregation Using iPS and Derived Megakaryocytes | Platelet Aggregation | Prospective Longitudinal Cohort | 250 | 125 | [phs001074.v1.p1.c2](https://www.ncbi.nlm.nih.gov/projects/gap/cgi-bin/study.cgi?study_id=phs001074.v1.p1) |
| GENESTAR | NHLBI TOPMed: Genetic Study of Atherosclerosis Risk (GeneSTAR) | Platelet Aggregation | Prospective Longitudinal Cohort | 1787 | 157 | [phs001218.v3.p1.c2](https://www.ncbi.nlm.nih.gov/projects/gap/cgi-bin/study.cgi?study_id=phs001218.v3.p1) |
| GENOA | Genetic Epidemiology Network of Arteriopathy (GENOA) | Hypertension | Prospective Longitudinal Cohort / Family/Twin/Trios | 3462 | 996 | [phs001238.v2.p1.c1](https://www.ncbi.nlm.nih.gov/projects/gap/cgi-bin/study.cgi?study_id=phs001238.v2.p1) |
| GENOA | NHLBI TOPMed: Genetic Epidemiology Network of Arteriopathy (GENOA) | Hypertension | Prospective Longitudinal Cohort / Family/Twin/Trios | 1854 | 16 | [phs001345.v3.p1.c1](https://www.ncbi.nlm.nih.gov/projects/gap/cgi-bin/study.cgi?study_id=phs001345.v3.p1) |
| GENSALT | Genetic Epidemiology Network of Salt Sensitivity (GenSalt) | Arterial Pressure | Interventional / Family/Twin/Trios | 1675 | 22 | [phs000784.v3.p1.c1](https://www.ncbi.nlm.nih.gov/projects/gap/cgi-bin/study.cgi?study_id=phs000784.v3.p1) |
| GENSALT | NHLBI TOPMed: Genetic Epidemiology Network of Salt Sensitivity (GenSalt) | Arterial Pressure | Interventional / Family/Twin/Trios | 3142 | 28 | [phs001217.v3.p1.c1](https://www.ncbi.nlm.nih.gov/projects/gap/cgi-bin/study.cgi?study_id=phs001217.v3.p1) |
| GGAF | NHLBI TOPMed - NHGRI CCDG: Groningen Genetics of Atrial Fibrillation (GGAF) Study | Atrial Fibrillation | Prospective Longitudinal Cohort | 640 | 14 | [phs001725.v3.p1.c1](https://www.ncbi.nlm.nih.gov/projects/gap/cgi-bin/study.cgi?study_id=phs001725.v3.p1) |
| GOLDN | NHLBI TOPMed: GOLDN Epigenetic Determinants of Lipid Response to Dietary Fat and Fenofibrate | Lipids | Prospective Longitudinal Cohort | 1069 | 17 | [phs001359.v3.p1.c1](https://www.ncbi.nlm.nih.gov/projects/gap/cgi-bin/study.cgi?study_id=phs001359.v3.p1) |
| HCHSSOL | Collaborative Cohort of Cohorts for COVID-19 Research (C4R): Hispanic Community Health Study/Study of Latinos (HCHS/SOL) |  | Prospective Longitudinal Cohort | 9368 | 273 | [phs002908.v1.p1.c2](https://www.ncbi.nlm.nih.gov/projects/gap/cgi-bin/study.cgi?study_id=phs002908.v1.p1) |
| HCHSSOL | Collaborative Cohort of Cohorts for COVID-19 Research (C4R): Hispanic Community Health Study/Study of Latinos (HCHS/SOL) |  | Prospective Longitudinal Cohort | 1814 | 273 | [phs002908.v1.p1.c1](https://www.ncbi.nlm.nih.gov/projects/gap/cgi-bin/study.cgi?study_id=phs002908.v1.p1) |
| HCHSSOL | Hispanic Community Health Study /Study of Latinos (HCHS/SOL) | Cardiovascular Diseases | Prospective Longitudinal Cohort | 3681 | 274 | [phs000810.v1.p1.c1](https://www.ncbi.nlm.nih.gov/projects/gap/cgi-bin/study.cgi?study_id=phs000810.v1.p1) |
| HCHSSOL | Hispanic Community Health Study /Study of Latinos (HCHS/SOL) | Cardiovascular Diseases | Prospective Longitudinal Cohort | 9214 | 274 | [phs000810.v1.p1.c2](https://www.ncbi.nlm.nih.gov/projects/gap/cgi-bin/study.cgi?study_id=phs000810.v1.p1) |
| HCHSSOL | NHLBI TOPMed: NHGRI CCDG: Hispanic Community Health Study/Study of Latinos (HCHS/SOL) | Cardiovascular Diseases | Prospective Longitudinal Cohort | 1270 | 13 | [phs001395.v2.p1.c1](https://www.ncbi.nlm.nih.gov/projects/gap/cgi-bin/study.cgi?study_id=phs001395.v2.p1) |
| HCHSSOL | NHLBI TOPMed: NHGRI CCDG: Hispanic Community Health Study/Study of Latinos (HCHS/SOL) | Cardiovascular Diseases | Prospective Longitudinal Cohort | 6823 | 13 | [phs001395.v2.p1.c2](https://www.ncbi.nlm.nih.gov/projects/gap/cgi-bin/study.cgi?study_id=phs001395.v2.p1) |
| HCT_for_SCD | Hematopoietic Cell Transplant for Sickle Cell Disease (HCT for SCD) | Anemia, Sickle Cell | Prospective Longitudinal Cohort | 1518 | 157 | [phs002385.v1.p1.c1](https://www.ncbi.nlm.nih.gov/projects/gap/cgi-bin/study.cgi?study_id=phs002385.v1.p1) |
| HF_Collection_ARDSnet_gen | ARDSnet and the iSPAAR Consortium: Genomic Basis of Susceptibility and Outcomes in Patients with the Acute Respiratory Distress Syndrome (ARDS) |  | Clinical Trial | 3033 | 22 | [phs000631.v1.p1.c1](https://www.ncbi.nlm.nih.gov/projects/gap/cgi-bin/study.cgi?study_id=phs000631.v1.p1) |
| HFN_DOSE_AHF | Heart Failure Network: Diuretic Optimization Strategies Evaluation in Acute Heart Failure (HFN DOSE-BioLINCC) | Heart Failure | Clinical Trial | 308 | 192 | [phs003524.v1.p1.c1](https://www.ncbi.nlm.nih.gov/projects/gap/cgi-bin/study.cgi?study_id=phs003524.v1.p1) |
| HFN_NEAT-BioLINCC | Heart Failure Network - Nitrate's Effect on Activity Tolerance in Heart Failure with Preserved Ejection Fraction (HFN NEAT-BioLINCC) |  | Clinical Trial | 110 | 265 | [phs003548.v1.p1.c1](https://www.ncbi.nlm.nih.gov/projects/gap/cgi-bin/study.cgi?study_id=phs003548.v1.p1) |
| HFN-EXACT-Biolincc | Xanthine Oxidase Inhibition for Hyperuricemic Heart Failure Patients (EXACT HF) | Heart Failure | Clinical Trial | 253 | 266 | [phs003533.v1.p1.c1](https://www.ncbi.nlm.nih.gov/projects/gap/cgi-bin/study.cgi?study_id=phs003533.v1.p1) |
| HLC | Human Liver Cohort | Liver | Cross-Sectional | 228 | 14 | [phs000253.v1.p1.c1](https://www.ncbi.nlm.nih.gov/projects/gap/cgi-bin/study.cgi?study_id=phs000253.v1.p1) |
| HVH | Heart and Vascular Health Study (HVH) | Cardiovascular Diseases | Case-Control (clinical) and case set (genomic) | 25 | 38 | [phs001013.v3.p2.c2](https://www.ncbi.nlm.nih.gov/projects/gap/cgi-bin/study.cgi?study_id=phs001013.v3.p2) |
| HVH | Heart and Vascular Health Study (HVH) | Cardiovascular Diseases | Case-Control (clinical) and case set (genomic) | 1179 | 38 | [phs001013.v3.p2.c1](https://www.ncbi.nlm.nih.gov/projects/gap/cgi-bin/study.cgi?study_id=phs001013.v3.p2) |
| HVH | NHLBI TOPMed: Heart and Vascular Health Study (HVH) | Cardiovascular Diseases | Case-Control (clinical) and case set (genomic) | 697 | 11 | [phs000993.v5.p2.c1](https://www.ncbi.nlm.nih.gov/projects/gap/cgi-bin/study.cgi?study_id=phs000993.v5.p2) |
| HVH | NHLBI TOPMed: Heart and Vascular Health Study (HVH) | Cardiovascular Diseases | Case-Control (clinical) and case set (genomic) | 12 | 11 | [phs000993.v5.p2.c2](https://www.ncbi.nlm.nih.gov/projects/gap/cgi-bin/study.cgi?study_id=phs000993.v5.p2) |
| HYPERGEN | NHLBI TOPMed: HyperGEN - Genetics of Left Ventricular (LV) Hypertrophy | Hypertrophy, Left Ventricular | Family/Twin/Trios | 330 | 164 | [phs001293.v3.p1.c2](https://www.ncbi.nlm.nih.gov/projects/gap/cgi-bin/study.cgi?study_id=phs001293.v3.p1) |
| HYPERGEN | NHLBI TOPMed: HyperGEN - Genetics of Left Ventricular (LV) Hypertrophy | Hypertrophy, Left Ventricular | Family/Twin/Trios | 1774 | 164 | [phs001293.v3.p1.c1](https://www.ncbi.nlm.nih.gov/projects/gap/cgi-bin/study.cgi?study_id=phs001293.v3.p1) |
| Imaging_CHS | Cardiovascular Health Study (CHS) Imaging | Cardiovascular Diseases | Prospective Longitudinal Cohort | 5 | 902 | [phs003639.v1.p1.c3](https://www.ncbi.nlm.nih.gov/projects/gap/cgi-bin/study.cgi?study_id=phs003639.v1.p1) |
| Imaging_CHS | Cardiovascular Health Study (CHS) Imaging | Cardiovascular Diseases | Prospective Longitudinal Cohort | 7 | 902 | [phs003639.v1.p1.c4](https://www.ncbi.nlm.nih.gov/projects/gap/cgi-bin/study.cgi?study_id=phs003639.v1.p1) |
| Imaging_CHS | Cardiovascular Health Study (CHS) Imaging | Cardiovascular Diseases | Prospective Longitudinal Cohort | 174 | 902 | [phs003639.v1.p1.c2](https://www.ncbi.nlm.nih.gov/projects/gap/cgi-bin/study.cgi?study_id=phs003639.v1.p1) |
| Imaging_CHS | Cardiovascular Health Study (CHS) Imaging | Cardiovascular Diseases | Prospective Longitudinal Cohort | 5353 | 902 | [phs003639.v1.p1.c1](https://www.ncbi.nlm.nih.gov/projects/gap/cgi-bin/study.cgi?study_id=phs003639.v1.p1) |
| Imaging_FHS | Framingham Heart Study-Cohort (FHS-Cohort) - Imaging | Longitudinal Studies | Prospective Longitudinal Cohort | 13169 | 386 | [phs003593.v1.p1.c1](https://www.ncbi.nlm.nih.gov/projects/gap/cgi-bin/study.cgi?study_id=phs003593.v1.p1) |
| Imaging_FHS | Framingham Heart Study-Cohort (FHS-Cohort) - Imaging | Longitudinal Studies | Prospective Longitudinal Cohort | 2279 | 386 | [phs003593.v1.p1.c2](https://www.ncbi.nlm.nih.gov/projects/gap/cgi-bin/study.cgi?study_id=phs003593.v1.p1) |
| Imaging_HFN_INDIE | Inorganic Nitrite Delivery to Improve Exercise Capacity in Heart Failure with Preserved Ejection Fraction (INDIE-HFpEF): Heart Failure Network (HFN INDIE-Imaging) |  | Prospective Longitudinal Cohort | 100 | 6 | [phs003804.v1.p1.c1](https://www.ncbi.nlm.nih.gov/projects/gap/cgi-bin/study.cgi?study_id=phs003804.v1.p1) |
| Imaging_JHS | Jackson Heart Study (JHS) Imaging |  | Collection | 711 | 6 | [phs003747.v1.p1.c2](https://www.ncbi.nlm.nih.gov/projects/gap/cgi-bin/study.cgi?study_id=phs003747.v1.p1) |
| Imaging_JHS | Jackson Heart Study (JHS) Imaging |  | Collection | 201 | 6 | [phs003747.v1.p1.c4](https://www.ncbi.nlm.nih.gov/projects/gap/cgi-bin/study.cgi?study_id=phs003747.v1.p1) |
| Imaging_JHS | Jackson Heart Study (JHS) Imaging |  | Collection | 404 | 6 | [phs003747.v1.p1.c3](https://www.ncbi.nlm.nih.gov/projects/gap/cgi-bin/study.cgi?study_id=phs003747.v1.p1) |
| Imaging_JHS | Jackson Heart Study (JHS) Imaging |  | Collection | 2567 | 6 | [phs003747.v1.p1.c1](https://www.ncbi.nlm.nih.gov/projects/gap/cgi-bin/study.cgi?study_id=phs003747.v1.p1) |
| Imaging_MESA_ECG | Multi-Ethnic Study of Atherosclerosis (Electrocardiogram Tracing Repository) | Atherosclerosis | Prospective Longitudinal Cohort | 771 | 92 | [phs003703.v1.p1.c2](https://www.ncbi.nlm.nih.gov/projects/gap/cgi-bin/study.cgi?study_id=phs003703.v1.p1) |
| Imaging_MESA_ECG | Multi-Ethnic Study of Atherosclerosis (Electrocardiogram Tracing Repository) | Atherosclerosis | Prospective Longitudinal Cohort | 6043 | 92 | [phs003703.v1.p1.c1](https://www.ncbi.nlm.nih.gov/projects/gap/cgi-bin/study.cgi?study_id=phs003703.v1.p1) |
| Imaging_MESA_ECHO | Multi-Ethnic Study of Atherosclerosis (Echocardiogram Image Repository) |  | Prospective Longitudinal Cohort | 6043 | 7 | [phs003702.v1.p1.c1](https://www.ncbi.nlm.nih.gov/projects/gap/cgi-bin/study.cgi?study_id=phs003702.v1.p1) |
| Imaging_MESA_ECHO | Multi-Ethnic Study of Atherosclerosis (Echocardiogram Image Repository) |  | Prospective Longitudinal Cohort | 771 | 7 | [phs003702.v1.p1.c2](https://www.ncbi.nlm.nih.gov/projects/gap/cgi-bin/study.cgi?study_id=phs003702.v1.p1) |
| Imaging_WHI | Women's Health Initiative Clinical Trial and Observational Study - Imaging |  | Clinical Trial | 59668 | 195 | [phs003824.v1.p1.c1](https://www.ncbi.nlm.nih.gov/projects/gap/cgi-bin/study.cgi?study_id=phs003824.v1.p1) |
| Imaging_WHI | Women's Health Initiative Clinical Trial and Observational Study - Imaging |  | Clinical Trial | 8311 | 195 | [phs003824.v1.p1.c2](https://www.ncbi.nlm.nih.gov/projects/gap/cgi-bin/study.cgi?study_id=phs003824.v1.p1) |
| INSPIRE_AF | NHLBI TOPMed: NHGRI CCDG: Intermountain INSPIRE Registry | Atrial Fibrillation | Case Set | 476 | 18 | [phs001545.v2.p1.c1](https://www.ncbi.nlm.nih.gov/projects/gap/cgi-bin/study.cgi?study_id=phs001545.v2.p1) |
| IPF | NHLBI TOPMed: Pulmonary Fibrosis Whole Genome Sequencing | Idiopathic Pulmonary Fibrosis | Case Set | 97 | 24 | [phs001607.v4.p3.c3](https://www.ncbi.nlm.nih.gov/projects/gap/cgi-bin/study.cgi?study_id=phs001607.v4.p3) |
| IPF | NHLBI TOPMed: Pulmonary Fibrosis Whole Genome Sequencing | Idiopathic Pulmonary Fibrosis | Case Set | 480 | 24 | [phs001607.v4.p3.c5](https://www.ncbi.nlm.nih.gov/projects/gap/cgi-bin/study.cgi?study_id=phs001607.v4.p3) |
| IPF | NHLBI TOPMed: Pulmonary Fibrosis Whole Genome Sequencing | Idiopathic Pulmonary Fibrosis | Case Set | 14 | 24 | [phs001607.v4.p3.c4](https://www.ncbi.nlm.nih.gov/projects/gap/cgi-bin/study.cgi?study_id=phs001607.v4.p3) |
| IPF | NHLBI TOPMed: Pulmonary Fibrosis Whole Genome Sequencing | Idiopathic Pulmonary Fibrosis | Case Set | 356 | 24 | [phs001607.v4.p3.c1](https://www.ncbi.nlm.nih.gov/projects/gap/cgi-bin/study.cgi?study_id=phs001607.v4.p3) |
| IPF | NHLBI TOPMed: Pulmonary Fibrosis Whole Genome Sequencing | Idiopathic Pulmonary Fibrosis | Case Set | 2388 | 24 | [phs001607.v4.p3.c2](https://www.ncbi.nlm.nih.gov/projects/gap/cgi-bin/study.cgi?study_id=phs001607.v4.p3) |
| IPF | NHLBI TOPMed: Pulmonary Fibrosis Whole Genome Sequencing | Idiopathic Pulmonary Fibrosis | Case Set | 361 | 24 | [phs001607.v4.p3.c6](https://www.ncbi.nlm.nih.gov/projects/gap/cgi-bin/study.cgi?study_id=phs001607.v4.p3) |
| JHS | NHLBI TOPMed: The Jackson Heart Study | Cardiovascular Diseases | Prospective Longitudinal Cohort | 180 | 24 | [phs000964.v5.p1.c2](https://www.ncbi.nlm.nih.gov/projects/gap/cgi-bin/study.cgi?study_id=phs000964.v5.p1) |
| JHS | NHLBI TOPMed: The Jackson Heart Study | Cardiovascular Diseases | Prospective Longitudinal Cohort | 797 | 24 | [phs000964.v5.p1.c1](https://www.ncbi.nlm.nih.gov/projects/gap/cgi-bin/study.cgi?study_id=phs000964.v5.p1) |
| JHS | NHLBI TOPMed: The Jackson Heart Study | Cardiovascular Diseases | Prospective Longitudinal Cohort | 2131 | 24 | [phs000964.v5.p1.c3](https://www.ncbi.nlm.nih.gov/projects/gap/cgi-bin/study.cgi?study_id=phs000964.v5.p1) |
| JHS | NHLBI TOPMed: The Jackson Heart Study | Cardiovascular Diseases | Prospective Longitudinal Cohort | 488 | 24 | [phs000964.v5.p1.c4](https://www.ncbi.nlm.nih.gov/projects/gap/cgi-bin/study.cgi?study_id=phs000964.v5.p1) |
| JHU_AF | NHLBI TOPMed - NHGRI CCDG: The Johns Hopkins University School of Medicine Atrial Fibrillation Genetics Study | Atrial Fibrillation | Case Set | 290 | 18 | [phs001598.v3.p1.c1](https://www.ncbi.nlm.nih.gov/projects/gap/cgi-bin/study.cgi?study_id=phs001598.v3.p1) |
| LMD-FSS | Genetic Analysis of Limb Malformation Disorders: Freeman Sheldon Syndrome Exome Sequencing Study (LMD-FSS) |  | Exome Sequencing | 3 | 9 | [phs000204.v1.p1.c1](https://www.ncbi.nlm.nih.gov/projects/gap/cgi-bin/study.cgi?study_id=phs000204.v1.p1) |
| LMD-MS | Genetic Analysis of Limb Malformation Disorders: Miller Syndrome Sequencing Study (LMD-MS) |  | Exome Sequencing | 6 | 14 | [phs000244.v1.p1.c1](https://www.ncbi.nlm.nih.gov/projects/gap/cgi-bin/study.cgi?study_id=phs000244.v1.p1) |
| LTRC | NHLBI TOPMed: Lung Tissue Research Consortium (LTRC) | Pulmonary Disease, Chronic Obstructive | Case-Control | 1602 | 1378 | [phs001662.v3.p1.c2](https://www.ncbi.nlm.nih.gov/projects/gap/cgi-bin/study.cgi?study_id=phs001662.v3.p1) |
| LungMAP-MALD | LungMAP: Molecular Atlas of Lung Development - Human Lung Tissue | Lung | Control Set | 54 | 17 | [phs001961.v3.p1.c1](https://www.ncbi.nlm.nih.gov/projects/gap/cgi-bin/study.cgi?study_id=phs001961.v3.p1) |
| MAGNet | Myocardial Applied Genomics Network (MAGNet) Study | Health | Case-Control | 151 | 14 | [phs001539.v4.p1.c1](https://www.ncbi.nlm.nih.gov/projects/gap/cgi-bin/study.cgi?study_id=phs001539.v4.p1) |
| MASALA | The Mediators of Atherosclerosis in South Asians Living in America (MASALA) - The Collaborative Cohort of Cohorts for COVID-19 Research (C4R) | Asian Americans | Prospective Longitudinal Cohort | 571 | 513 | [phs002980.v1.p1.c1](https://www.ncbi.nlm.nih.gov/projects/gap/cgi-bin/study.cgi?study_id=phs002980.v1.p1) |
| MAYOVTE | NHLBI TOPMed: Whole Genome Sequencing of Venous Thromboembolism (WGS of VTE) | Venous Thromboembolism | Case Set | 1535 | 52 | [phs001402.v3.p1.c1](https://www.ncbi.nlm.nih.gov/projects/gap/cgi-bin/study.cgi?study_id=phs001402.v2.p1) |
| MESA | Collaborative Cohort of Cohorts for COVID-19 Research (C4R): Multi-Ethnic Study of Atherosclerosis (MESA) | COVID-19 | Prospective Longitudinal Cohort | 294 | 574 | [phs003017.v1.p1.c2](https://www.ncbi.nlm.nih.gov/projects/gap/cgi-bin/study.cgi?study_id=phs003017.v1.p1) |
| MESA | Collaborative Cohort of Cohorts for COVID-19 Research (C4R): Multi-Ethnic Study of Atherosclerosis (MESA) | COVID-19 | Prospective Longitudinal Cohort | 3043 | 574 | [phs003017.v1.p1.c1](https://www.ncbi.nlm.nih.gov/projects/gap/cgi-bin/study.cgi?study_id=phs003017.v1.p1) |
| MESA | Multi-Ethnic Study of Atherosclerosis (MESA) SHARe | Cardiovascular Diseases | Prospective Longitudinal Cohort | 856 | 20088 | [phs000209.v13.p3.c2](https://www.ncbi.nlm.nih.gov/projects/gap/cgi-bin/study.cgi?study_id=phs000209.v13.p3) |
| MESA | Multi-Ethnic Study of Atherosclerosis (MESA) SHARe | Cardiovascular Diseases | Prospective Longitudinal Cohort | 7439 | 20088 | [phs000209.v13.p3.c1](https://www.ncbi.nlm.nih.gov/projects/gap/cgi-bin/study.cgi?study_id=phs000209.v13.p3) |
| MESA | NHLBI TOPMed: MESA and MESA Family AA-CAC | Cardiovascular Diseases | Prospective Longitudinal Cohort | 820 | 97 | [phs001416.v4.p1.c2](https://www.ncbi.nlm.nih.gov/projects/gap/cgi-bin/study.cgi?study_id=phs001416.v4.p1) |
| MESA | NHLBI TOPMed: MESA and MESA Family AA-CAC | Cardiovascular Diseases | Prospective Longitudinal Cohort | 7068 | 97 | [phs001416.v4.p1.c1](https://www.ncbi.nlm.nih.gov/projects/gap/cgi-bin/study.cgi?study_id=phs001416.v4.p1) |
| MGHAF | Massachusetts General Hospital (MGH) Atrial Fibrillation Study | Atrial Fibrillation | Case Set | 933 | 35 | [phs001001.v1.p1.c1](https://www.ncbi.nlm.nih.gov/projects/gap/cgi-bin/study.cgi?study_id=phs001001.v1.p1) |
| MGHAF | Massachusetts General Hospital (MGH) Atrial Fibrillation Study | Atrial Fibrillation | Case Set | 92 | 35 | [phs001001.v1.p1.c2](https://www.ncbi.nlm.nih.gov/projects/gap/cgi-bin/study.cgi?study_id=phs001001.v1.p1) |
| MGHAF | NHLBI TOPMed - NHGRI CCDG: Massachusetts General Hospital (MGH) Atrial Fibrillation Study | Atrial Fibrillation | Case Set | 908 | 16 | [phs001062.v5.p2.c1](https://www.ncbi.nlm.nih.gov/projects/gap/cgi-bin/study.cgi?study_id=phs001062.v5.p2) |
| MGHAF | NHLBI TOPMed - NHGRI CCDG: Massachusetts General Hospital (MGH) Atrial Fibrillation Study | Atrial Fibrillation | Case Set | 255 | 16 | [phs001062.v5.p2.c2](https://www.ncbi.nlm.nih.gov/projects/gap/cgi-bin/study.cgi?study_id=phs001062.v5.p2) |
| MIGen_ExS | Myocardial Infarction Genetics Exome Sequencing Consortium: BioImage Study |  | Case-Control | 503 | 10 | [phs001058.v1.p1.c1](https://www.ncbi.nlm.nih.gov/projects/gap/cgi-bin/study.cgi?study_id=phs001058.v1.p1) |
| miRhythm | NHLBI TOPMed: Defining the Time-Dependent Genetic and Transcriptomic Responses to Cardiac Injury Among Patients with Arrhythmias | Atrial Fibrillation | Case Set | 65 | 31 | [phs001434.v2.p1.c1](https://www.ncbi.nlm.nih.gov/projects/gap/cgi-bin/study.cgi?study_id=phs001434.v2.p1) |
| miRNA_Maternal_Plasma | miRNA Profiling of Maternal and Non-Maternal Healthy Adult Blood Plasma Using Small RNA-Sequencing | MicroRNAs | Case-Control | 13 | 18 | [phs001892.v1.p1.c1](https://www.ncbi.nlm.nih.gov/projects/gap/cgi-bin/study.cgi?study_id=phs001892.v1.p1) |
| MLOF | NHLBI TOPMed: My Life Our Future (MLOF) Research Repository of Patients with Hemophilia A (Factor VIII Deficiency) or Hemophilia B (Factor IX Deficiency) | Hemophilia A | Cross-Sectional | 9104 | 21 | [phs001515.v2.p2.c1](https://www.ncbi.nlm.nih.gov/projects/gap/cgi-bin/study.cgi?study_id=phs001515.v2.p2) |
| MolGen_CHD | Molecular Genetics of Heterotaxy and Related Congenital Heart Defects | Heterotaxy Syndrome | Case Set | 279 | 55 | [phs001814.v1.p1.c1](https://www.ncbi.nlm.nih.gov/projects/gap/cgi-bin/study.cgi?study_id=phs001814.v1.p1) |
| MPP | NHLBI TOPMed - NHGRI CCDG: Malmo Preventive Project (MPP) | Atrial Fibrillation | Case Set | 121 | 18 | [phs001544.v3.p1.c1](https://www.ncbi.nlm.nih.gov/projects/gap/cgi-bin/study.cgi?study_id=phs001544.v3.p1) |
| MSH | Multicenter Study of Hydroxyurea (MSH) | Anemia, Sickle Cell | Clinical Trial | 357 | 1725 | [phs002348.v1.p1.c1](https://www.ncbi.nlm.nih.gov/projects/gap/cgi-bin/study.cgi?study_id=phs002348.v1.p1) |
| NOMAS | Northern Manhattan Study (NOMAS) - The Collaborative Cohort of Cohorts for COVID-19 Research (C4R) | COVID-19 | Prospective Longitudinal Cohort | 995 | 458 | [phs003028.v1.p1.c1](https://www.ncbi.nlm.nih.gov/projects/gap/cgi-bin/study.cgi?study_id=phs003028.v1.p1) |
| NSRR_CFS | National Sleep Research Resource (NSRR): Cleveland Family Study (CFS) | Sleep Apnea Syndromes | Prospective Longitudinal Cohort | 710 | 2632 | [phs002715.v1.p1.c1](https://www.ncbi.nlm.nih.gov/projects/gap/cgi-bin/study.cgi?study_id=phs002715.v1.p1) |
| NSRR_HCHS | National Sleep Research Resource (NSRR) Hispanic Community Health Study (HCHS) | Cardiovascular Diseases | Prospective Longitudinal Cohort | 2304 | 492 | [phs003543.v1.p1.c1](https://www.ncbi.nlm.nih.gov/projects/gap/cgi-bin/study.cgi?study_id=phs003543.v1.p1) |
| NSRR_HCHS | National Sleep Research Resource (NSRR) Hispanic Community Health Study (HCHS) | Cardiovascular Diseases | Prospective Longitudinal Cohort | 9817 | 492 | [phs003543.v1.p1.c2](https://www.ncbi.nlm.nih.gov/projects/gap/cgi-bin/study.cgi?study_id=phs003543.v1.p1) |
| nuMoM2b | Nulliparous Pregnancy Outcomes Study: Monitoring Mothers-to-be Heart Health Study (nuMoM2b Heart Health Study) | Hypertension | Prospective Longitudinal Cohort | 8882 | 4476 | [phs002808.v1.p1.c1](https://www.ncbi.nlm.nih.gov/projects/gap/cgi-bin/study.cgi?study_id=phs002808.v1.p1) |
| OMG_SCD | NHLBI TOPMed: Outcome Modifying Genes in Sickle Cell Disease (OMG) | Anemia, Sickle Cell | Case Set | 642 | 65 | [phs001608.v2.p1.c1](https://www.ncbi.nlm.nih.gov/projects/gap/cgi-bin/study.cgi?study_id=phs001608.v2.p1) |
| ORCHID | COVID19-ORCHID | COVID-19 | Clinical Trial | 479 | 754 | [phs002299.v1.p1.c1](https://www.ncbi.nlm.nih.gov/projects/gap/cgi-bin/study.cgi?study_id=phs002299.v1.p1) |
| PARTNERS | NHLBI TOPMed: Partners HealthCare Biobank | Atrial Fibrillation | Case Set | 128 | 32 | [phs001024.v6.p1.c1](https://www.ncbi.nlm.nih.gov/projects/gap/cgi-bin/study.cgi?study_id=phs001024.v6.p1) |
| PCGC | Congenital Heart Disease Genetic Network Study | Heart Defects, Congenital | Prospective Longitudinal Cohort | 12846 | 8 | [phs000571.v6.p2.c1](https://www.ncbi.nlm.nih.gov/projects/gap/cgi-bin/study.cgi?study_id=phs000571.v6.p2) |
| PCGC | Congenital Heart Disease Genetic Network Study | Heart Defects, Congenital | Prospective Longitudinal Cohort | 49 | 8 | [phs000571.v6.p2.c2](https://www.ncbi.nlm.nih.gov/projects/gap/cgi-bin/study.cgi?study_id=phs000571.v6.p2) |
| PCGC | The Pediatric Cardiac Genetics Consortium (PCGC) Study | Heart Defects, Congenital | Prospective Longitudinal Cohort | 32 | 434 | [phs001194.v2.p2.c2](https://www.ncbi.nlm.nih.gov/projects/gap/cgi-bin/study.cgi?study_id=phs001194.v2.p2) |
| PCGC | The Pediatric Cardiac Genetics Consortium (PCGC) Study | Heart Defects, Congenital | Prospective Longitudinal Cohort | 9431 | 434 | [phs001194.v2.p2.c1](https://www.ncbi.nlm.nih.gov/projects/gap/cgi-bin/study.cgi?study_id=phs001194.v2.p2) |
| PCGC_CHD | NHLBI TOPMed: Pediatric Cardiac Genomics Consortium (PCGC)'s Congenital Heart Disease Biobank | Heart Defects, Congenital | Prospective Longitudinal Cohort | 8 | 17 | [phs001735.v2.p1.c2](https://www.ncbi.nlm.nih.gov/projects/gap/cgi-bin/study.cgi?study_id=phs001735.v2.p1) |
| PCGC_CHD | NHLBI TOPMed: Pediatric Cardiac Genomics Consortium (PCGC)'s Congenital Heart Disease Biobank | Heart Defects, Congenital | Prospective Longitudinal Cohort | 4542 | 17 | [phs001735.v2.p1.c1](https://www.ncbi.nlm.nih.gov/projects/gap/cgi-bin/study.cgi?study_id=phs001735.v2.p1) |
| PharmHU | NHLBI TOPMed: Pharmacogenomics of Hydroxyurea in Sickle Cell Disease (PharmHU) | Anemia, Sickle Cell | Case Set | 58 | 22 | [phs001466.v2.p1.c2](https://www.ncbi.nlm.nih.gov/projects/gap/cgi-bin/study.cgi?study_id=phs001466.v2.p1) |
| PharmHU | NHLBI TOPMed: Pharmacogenomics of Hydroxyurea in Sickle Cell Disease (PharmHU) | Anemia, Sickle Cell | Case Set | 740 | 22 | [phs001466.v2.p1.c1](https://www.ncbi.nlm.nih.gov/projects/gap/cgi-bin/study.cgi?study_id=phs001466.v2.p1) |
| PharmHU | NHLBI TOPMed: Pharmacogenomics of Hydroxyurea in Sickle Cell Disease (PharmHU) | Anemia, Sickle Cell | Case Set | 102 | 22 | [phs001466.v2.p1.c3](https://www.ncbi.nlm.nih.gov/projects/gap/cgi-bin/study.cgi?study_id=phs001466.v2.p1) |
| PhLiPS | The National Heart, Lung, and Blood Institute (NHLBI)-funded Next Generation Genetic Association Studies (NextGen) Consortium: Phenotyping Lipid traits in iPS derived hepatocytes Study (PhLiPS Study) | Lipids | Prospective Longitudinal Cohort | 90 | 28 | [phs001341.v1.p1.c1](https://www.ncbi.nlm.nih.gov/projects/gap/cgi-bin/study.cgi?study_id=phs001341.v1.p1) |
| PIMA | NHLBI TOPMed: Pathways to Immunologically Mediated Asthma (PIMA) | Asthma | Prospective Longitudinal Cohort | 73 | 24 | [phs001727.v3.p1.c2](https://www.ncbi.nlm.nih.gov/projects/gap/cgi-bin/study.cgi?study_id=phs001727.v3.p1) |
| Prepf | Collaborative Cohort of Cohorts for COVID-19 Research (C4R): Prevent Pulmonary Fibrosis (PrePF) | COVID-19 | Prospective Longitudinal Cohort | 628 | 395 | [phs002975.v1.p1.c1](https://www.ncbi.nlm.nih.gov/projects/gap/cgi-bin/study.cgi?study_id=phs002975.v1.p1) |
| PROMIS | NHLBI TOPMed - NHGRI CCDG: Pakistan Risk of Myocardial Infarction Study (PROMIS) |  | Case-Control | 26061 | 22 | [phs001569.v1.p1.c1](https://www.ncbi.nlm.nih.gov/projects/gap/cgi-bin/study.cgi?study_id=phs001569.v1.p1) |
| PUSH_SCD | NHLBI TOPMed: Pulmonary Hypertension and the Hypoxic Response in SCD (PUSH) | Anemia, Sickle Cell | Case-Control | 432 | 84 | [phs001682.v2.p1.c1](https://www.ncbi.nlm.nih.gov/projects/gap/cgi-bin/study.cgi?study_id=phs001682.v2.p1) |
| RECOVER_Adult | Researching COVID to Enhance Recovery (RECOVER): Adult Observational Cohort Study | COVID-19 | Prospective Longitudinal Cohort | 15179 | 211053 | [phs003463.v4.p3.c1](https://www.ncbi.nlm.nih.gov/projects/gap/cgi-bin/study.cgi?study_id=phs003463.v4.p3) |
| RECOVER_Autopsy | NIH RECOVER: A Multi-Site Pathology Study of Post-Acute Sequelae of SARS-CoV-2 Infection |  | Prospective Longitudinal Cohort | 197 | 1954 | [phs003768.v1.p1.c1](https://www.ncbi.nlm.nih.gov/projects/gap/cgi-bin/study.cgi?study_id=phs003768.v1.p1) |
| RECOVER_Pediatric | Researching COVID to Enhance Recovery (RECOVER): Post Acute Sequelae of SARS-CoV-2 (PASC) Pediatric Cohort Study |  | Case Set | 25043 | 22152 | [phs003461.v2.p2.c1](https://www.ncbi.nlm.nih.gov/projects/gap/cgi-bin/study.cgi?study_id=phs003461.v2.p2) |
| RED_CORAL | PETAL Repository of Electronic Data COVID-19 Observational Study (RED CORAL) | COVID-19 | Control Set | 1480 | 1544 | [phs002363.v1.p1.c1](https://www.ncbi.nlm.nih.gov/projects/gap/cgi-bin/study.cgi?study_id=phs002363.v1.p1) |
| REDS-BSCDC | NHLBI TOPMed: Recipient Epidemiology and Donor Evaluation Study-III Brazil Sickle Cell Disease Cohort (REDS-BSCDC) | Sickle Cell Anemia | Prospective Longitudinal Cohort | 2795 | 25 | [phs001468.v3.p1.c1](https://www.ncbi.nlm.nih.gov/projects/gap/cgi-bin/study.cgi?study_id=phs001468.v3.p1) |
| REGARDS | REasons for Geographic and Racial Differences in Stroke (REGARDS) - The Collaborative Cohort of Cohorts for COVID-19 Research (C4R) | COVID-19 | Prospective Longitudinal Cohort | 8707 | 307 | [phs002919.v1.p1.c1](https://www.ncbi.nlm.nih.gov/projects/gap/cgi-bin/study.cgi?study_id=phs002919.v1.p1) |
| RESPONSE | REDS-IV-P Epidemiology, Surveillance and Preparedness of the Novel SARS-CoV-2 Epidemic | SARS-CoV-2 | Prospective Longitudinal Cohort | 4729 | 204 | [phs003578.v1.p1.c1](https://www.ncbi.nlm.nih.gov/projects/gap/cgi-bin/study.cgi?study_id=phs003578.v1.p1) |
| ROC-Cardiac_Epistry_3-BioLINCC | Resuscitation Outcomes Consortium (ROC) Cardiac Epidemiologic Registry (Cardiac Epistry) Version 3 (ROC-Cardiac Epistry 3-BioLINCC) |  | Prospective Longitudinal Cohort | 120306 | 424 | [phs003726.v1.p1.c1](https://www.ncbi.nlm.nih.gov/projects/gap/cgi-bin/study.cgi?study_id=phs003726.v1.p1) |
| SAFHS | NHLBI TOPMed: San Antonio Family Heart Study (SAFHS) | Cardiovascular Diseases | Family/Twin/Trios | 2594 | 46 | [phs001215.v4.p2.c1](https://www.ncbi.nlm.nih.gov/projects/gap/cgi-bin/study.cgi?study_id=phs001215.v3.p2) |
| SAGE | NHLBI TOPMed: Study of African Americans, Asthma, Genes and Environment (SAGE) Study | Lung Diseases | Case Set | 1964 | 33 | [phs000921.v5.p2.c2](https://www.ncbi.nlm.nih.gov/projects/gap/cgi-bin/study.cgi?study_id=phs000921.v5.p2) |
| SAPPHIRE | NHLBI TOPMed: Study of Asthma Phenotypes and Pharmacogenomic Interactions by Race-Ethnicity (SAPPHIRE) | Asthma | Prospective Longitudinal Cohort | 4857 | 32 | [phs001467.v2.p2.c1](https://www.ncbi.nlm.nih.gov/projects/gap/cgi-bin/study.cgi?study_id=phs001467.v2.p2) |
| SARCOIDOSIS | NHLBI TOPMed: African American Sarcoidosis Genetics Resource | Sarcoidosis | Family/Twin/Trios | 1335 | 30 | [phs001207.v3.p1.c1](https://www.ncbi.nlm.nih.gov/projects/gap/cgi-bin/study.cgi?study_id=phs001207.v3.p1) |
| SARP | NHLBI GO-ESP: Lung Cohorts Exome Sequencing Project (Asthma): Genetic variants affecting susceptibility and severity | Asthma | Case Set | 191 | 27 | [phs000422.v1.p1.c1](https://www.ncbi.nlm.nih.gov/projects/gap/cgi-bin/study.cgi?study_id=phs000422.v1.p1) |
| SARP | NHLBI TOPMed: Severe Asthma Research Program (SARP) | Asthma | Case Set | 671 | 42 | [phs001446.v3.p1.c2](https://www.ncbi.nlm.nih.gov/projects/gap/cgi-bin/study.cgi?study_id=phs001446.v3.p1) |
| SARP | NHLBI TOPMed: Severe Asthma Research Program (SARP) | Asthma | Case Set | 1211 | 42 | [phs001446.v3.p1.c1](https://www.ncbi.nlm.nih.gov/projects/gap/cgi-bin/study.cgi?study_id=phs001446.v3.p1) |
| SARP | Severe Asthma Research Program (SARP) - The Collaborative Cohort of Cohorts for COVID-19 Research (C4R) | COVID-19 | Case Set | 307 | 459 | [phs002913.v1.p1.c4](https://www.ncbi.nlm.nih.gov/projects/gap/cgi-bin/study.cgi?study_id=phs002913.v1.p1) |
| SARP | Severe Asthma Research Program (SARP) - The Collaborative Cohort of Cohorts for COVID-19 Research (C4R) | COVID-19 | Case Set | 30 | 459 | [phs002913.v1.p1.c1](https://www.ncbi.nlm.nih.gov/projects/gap/cgi-bin/study.cgi?study_id=phs002913.v1.p1) |
| SARP | Severe Asthma Research Program (SARP) - The Collaborative Cohort of Cohorts for COVID-19 Research (C4R) | COVID-19 | Case Set | 116 | 459 | [phs002913.v1.p1.c2](https://www.ncbi.nlm.nih.gov/projects/gap/cgi-bin/study.cgi?study_id=phs002913.v1.p1) |
| SARP | Severe Asthma Research Program (SARP) - The Collaborative Cohort of Cohorts for COVID-19 Research (C4R) | COVID-19 | Case Set | 26 | 459 | [phs002913.v1.p1.c3](https://www.ncbi.nlm.nih.gov/projects/gap/cgi-bin/study.cgi?study_id=phs002913.v1.p1) |
| SAS | Genome-Wide Association Study of Adiposity in Samoans | Obesity | Cross-Sectional | 3501 | 178 | [phs000914.v1.p1.c1](https://www.ncbi.nlm.nih.gov/projects/gap/cgi-bin/study.cgi?study_id=phs000914.v1.p1) |
| SAS | NHLBI TOPMed: Genome-wide Association Study of Adiposity in Samoans | Obesity | Cross-Sectional | 1332 | 13 | [phs000972.v5.p1.c1](https://www.ncbi.nlm.nih.gov/projects/gap/cgi-bin/study.cgi?study_id=phs000972.v5.p1) |
| SCD | NHLBI TOPMed: Boston-Brazil Sickle Cell Disease (SCD) Cohort | Anemia, Sickle Cell | Prospective Longitudinal Cohort | 943 | 32 | [phs001599.v1.p1.c1](https://www.ncbi.nlm.nih.gov/projects/gap/cgi-bin/study.cgi?study_id=phs001599.v1.p1) |
| SCD_NHDR | Sickle Cell Disease Natural History Data Resource (SCD NHDR) | Anemia, Sickle Cell | Prospective Longitudinal Cohort | 1792 | 1459 | [phs003529.v2.p2.c1](https://www.ncbi.nlm.nih.gov/projects/gap/cgi-bin/study.cgi?study_id=phs003529.v1.p1) |
| SCD-HeFT-BioLINCC | Sudden Cardiac Death in Heart Failure Trial (SCD-HeFT-BioLINCC) | Heart Failure | Clinical Trial | 2521 | 715 | [phs003654.v1.p1.c1](https://www.ncbi.nlm.nih.gov/projects/gap/cgi-bin/study.cgi?study_id=phs003654.v1.p1) |
| SCVI | NHLBI TOPMed: Stanford Cardiovascular Institute iPSC Biobank Study (SCVI) |  | Collection | 1249 | 5 | [phs002338.v1.p1.c1](https://www.ncbi.nlm.nih.gov/projects/gap/cgi-bin/study.cgi?study_id=phs002338.v1.p1) |
| SEA | SNPs and Extent of Atherosclerosis (SEA) Study |  | Case-Control | 1068 | 21 | [phs000349.v1.p1.c1](https://www.ncbi.nlm.nih.gov/projects/gap/cgi-bin/study.cgi?study_id=phs000349.v1.p1) |
| SHARP | SNP Health Association Asthma Resource Project | Asthma | Cross-Sectional | 4046 | 315 | [phs000166.v2.p1.c1](https://www.ncbi.nlm.nih.gov/projects/gap/cgi-bin/study.cgi?study_id=phs000166.v2.p1) |
| SPIROMICS | NHLBI TOPMed: SubPopulations and InteRmediate Outcome Measures In COPD Study (SPIROMICS) | Pulmonary Disease, Chronic Obstructive | Prospective Longitudinal Cohort | 3 | 15 | [phs001927.v1.p1.c1](https://www.ncbi.nlm.nih.gov/projects/gap/cgi-bin/study.cgi?study_id=phs001927.v1.p1) |
| SPIROMICS | NHLBI TOPMed: SubPopulations and InteRmediate Outcome Measures In COPD Study (SPIROMICS) | Pulmonary Disease, Chronic Obstructive | Prospective Longitudinal Cohort | 2099 | 15 | [phs001927.v1.p1.c4](https://www.ncbi.nlm.nih.gov/projects/gap/cgi-bin/study.cgi?study_id=phs001927.v1.p1) |
| SPIROMICS | NHLBI TOPMed: SubPopulations and InteRmediate Outcome Measures In COPD Study (SPIROMICS) | Pulmonary Disease, Chronic Obstructive | Prospective Longitudinal Cohort | 71 | 15 | [phs001927.v1.p1.c3](https://www.ncbi.nlm.nih.gov/projects/gap/cgi-bin/study.cgi?study_id=phs001927.v1.p1) |
| SPIROMICS | NHLBI TOPMed: SubPopulations and InteRmediate Outcome Measures In COPD Study (SPIROMICS) | Pulmonary Disease, Chronic Obstructive | Prospective Longitudinal Cohort | 133 | 15 | [phs001927.v1.p1.c2](https://www.ncbi.nlm.nih.gov/projects/gap/cgi-bin/study.cgi?study_id=phs001927.v1.p1) |
| SPRINT | Systolic Blood Pressure Intervention Trial (SPRINT-Imaging) | Hypertension | Clinical Trial | 9361 | 323 | [phs003566.v1.p1.c1](https://www.ncbi.nlm.nih.gov/projects/gap/cgi-bin/study.cgi?study_id=phs003566.v1.p1) |
| STICH-BioLINCC | Surgical Treatment for Ischemic Heart Failure (STICH-BioLINCC) |  | Clinical Trial | 2621 | 329 | [phs003493.v1.p1.c1](https://www.ncbi.nlm.nih.gov/projects/gap/cgi-bin/study.cgi?study_id=phs003493.v1.p1) |
| STOP-II | Optimizing Primary Stroke Prevention in Children with Sickle Cell Anemia (STOP II) | Not Provided | Clinical Trial | 79 | 1217 | [phs002386.v1.p1.c1](https://www.ncbi.nlm.nih.gov/projects/gap/cgi-bin/study.cgi?study_id=phs002386.v1.p1) |
| THRV | NHLBI TOPMed: Rare Variants for Hypertension in Taiwan Chinese (THRV) | Blood Pressure | Prospective Longitudinal Cohort | 2353 | 90 | [phs001387.v3.p1.c3](https://www.ncbi.nlm.nih.gov/projects/gap/cgi-bin/study.cgi?study_id=phs001387.v3.p1) |
| TOPMed_Harmonized | Trans-Omics for Precision Medicine (TOPMed) Harmonized Phenotypes |  |  | 207747 | 125 |  |
| Transcribe | TRanscriptomic ANalySis of left ventriCulaR gene Expression (TRANSCRibE) | Cardiovascular Diseases | Prospective Longitudinal Cohort | 79 | 19 | [phs001679.v1.p1.c1](https://www.ncbi.nlm.nih.gov/projects/gap/cgi-bin/study.cgi?study_id=phs001679.v1.p1) |
| Transcribe | TRanscriptomic ANalySis of left ventriCulaR gene Expression (TRANSCRibE) | Cardiovascular Diseases | Prospective Longitudinal Cohort | 40 | 19 | [phs001679.v1.p1.c2](https://www.ncbi.nlm.nih.gov/projects/gap/cgi-bin/study.cgi?study_id=phs001679.v1.p1) |
| UCSF | NHLBI TOPMed - NHGRI CCDG: UCSF Atrial Fibrillation Study | Atrial Fibrillation | Case Set | 113 | 21 | [phs001933.v2.p1.c1](https://www.ncbi.nlm.nih.gov/projects/gap/cgi-bin/study.cgi?study_id=phs001933.v2.p1) |
| UTMB_HeLP | Incentives and Case Management to Improve Cardiac Care: Healthy Lifestyle Program (HeLP) |  | Clinical Trial | 192 | 32 | [phs003737.v1.p1.c1](https://www.ncbi.nlm.nih.gov/projects/gap/cgi-bin/study.cgi?study_id=phs003737.v1.p1) |
| VAFAR | NHLBI TOPMed - NHGRI CCDG: The Vanderbilt AF Ablation Registry | Atrial Fibrillation | Case Set | 173 | 31 | [phs000997.v5.p2.c1](https://www.ncbi.nlm.nih.gov/projects/gap/cgi-bin/study.cgi?study_id=phs000997.v5.p2) |
| VUAF | NHLBI TOPMed: The Vanderbilt Atrial Fibrillation Registry (VU_AF) | Atrial Fibrillation | Case Set | 1134 | 37 | [phs001032.v6.p2.c1](https://www.ncbi.nlm.nih.gov/projects/gap/cgi-bin/study.cgi?study_id=phs001032.v6.p2) |
| walk_PHaSST | NHLBI TOPMed: Walk-PHaSST Sickle Cell Disease (SCD) | Anemia, Sickle Cell | Clinical Trial | 50 | 85 | [phs001514.v2.p1.c2](https://www.ncbi.nlm.nih.gov/projects/gap/cgi-bin/study.cgi?study_id=phs001514.v2.p1) |
| walk_PHaSST | NHLBI TOPMed: Walk-PHaSST Sickle Cell Disease (SCD) | Anemia, Sickle Cell | Clinical Trial | 395 | 85 | [phs001514.v2.p1.c1](https://www.ncbi.nlm.nih.gov/projects/gap/cgi-bin/study.cgi?study_id=phs001514.v2.p1) |
| Walk-PHaSST | Treatment of Pulmonary Hypertension and Sickle Cell Disease with Sildenafil Therapy (Walk-PHaSST) | Anemia, Sickle Cell | Clinical Trial | 720 | 1101 | [phs002383.v1.p1.c1](https://www.ncbi.nlm.nih.gov/projects/gap/cgi-bin/study.cgi?study_id=phs002383.v1.p1) |
| WGHS | NHLBI TOPMed: Novel Risk Factors for the Development of Atrial Fibrillation in Women | Atrial Fibrillation | Case Set | 118 | 61 | [phs001040.v6.p1.c1](https://www.ncbi.nlm.nih.gov/projects/gap/cgi-bin/study.cgi?study_id=phs001040.v6.p1) |
| WHI | NHLBI TOPMed: Women's Health Initiative (WHI) | Stroke | Prospective Longitudinal Cohort | 2314 | 98 | [phs001237.v4.p2.c2](https://www.ncbi.nlm.nih.gov/projects/gap/cgi-bin/study.cgi?study_id=phs001237.v4.p2) |
| WHI | NHLBI TOPMed: Women's Health Initiative (WHI) | Stroke | Prospective Longitudinal Cohort | 10793 | 98 | [phs001237.v4.p2.c1](https://www.ncbi.nlm.nih.gov/projects/gap/cgi-bin/study.cgi?study_id=phs001237.v4.p2) |
| WHI | Women's Health Initiative Clinical Trial and Observational Study | Women's Health | Prospective Longitudinal Cohort | 117675 | 6154 | [phs000200.v12.p3.c1](https://www.ncbi.nlm.nih.gov/projects/gap/cgi-bin/study.cgi?study_id=phs000200.v12.p3) |
| WHI | Women's Health Initiative Clinical Trial and Observational Study | Women's Health | Prospective Longitudinal Cohort | 25538 | 6154 | [phs000200.v12.p3.c2](https://www.ncbi.nlm.nih.gov/projects/gap/cgi-bin/study.cgi?study_id=phs000200.v12.p3) |

**Supplementary Table 2. Comparison of Open and Authorized PIC-SURE**

| **Feature** | **Open PIC-SURE** | **Authorized PIC-SURE** |
| --- | --- | --- |
| Publicly available | ✓ |  |
| Some variables not available for filtering* | ✓ |  |
| Data obfuscation | ✓ |  |
| Authorization to access data required |  | ✓ |
| Search on participant-level data | ✓ | ✓ |
| Returns aggregate counts | ✓ | ✓ |
| Access to participant-level data |  | ✓ |
| Phenotypic variable search | ✓ | ✓ |
| Phenotypic variable filtering | ✓ | ✓ |
| Genomic variable filtering |  | ✓ |
| Participant data retrieval |  | ✓ |
| Visualizations | ✓ | ✓ |

* Stigmatizing variables and other variables are removed from Open PIC-SURE to protect participant anonymity.
